# Cranial Morphology of the Late Oligocene Patagonian Notohippid *Rhynchippus equinus* Ameghino, 1897 (Mammalia, Notoungulata) with Emphases in Basicranial and Auditory Region

**DOI:** 10.1371/journal.pone.0156558

**Published:** 2016-05-27

**Authors:** Gastón Martínez, María Teresa Dozo, Javier N. Gelfo, Hernán Marani

**Affiliations:** 1 Instituto Patagónico de Geología y Paleontología, Centro Nacional Patagónico, CONICET, Puerto Madryn, Chubut, Argentina; 2 Facultad de Ciencias Exactas Físicas y Naturales, Universidad Nacional de Córdoba, Córdoba, Argentina; 3 División Paleontología Vertebrados, Museo de la Plata, CONICET, La Plata, Buenos Aires, Argentina; 4 Facultad de Ciencias Naturales y Museo, Universidad Nacional de La Plata, La Plata, Buenos Aires, Argentina; 5 Facultad de Ciencias Naturales, Universidad Nacional de la Patagonia San Juan Bosco, Puerto Madryn, Chubut, Argentina; Institute of Vertebrate Paleontology and Paleoanthropology Chinese Academy of Sciences, CHINA

## Abstract

“Notohippidae” is a probably paraphyletic family of medium sized notoungulates with complete dentition and early tendency to hypsodonty. They have been recorded from early Eocene to early Miocene, being particularly diverse by the late Oligocene. Although *Rhynchippus equinus* Ameghino is one of the most frequent notohippids in the fossil record, there are scarce data about cranial osteology other than the classical descriptions which date back to the early last century. In this context, we describe the exceptionally preserved specimen MPEF PV 695 (based on CT scanning technique and 3D reconstruction) with the aim of improving our knowledge of the species, especially regarding auditory region (petrosal, tympanic and surrounding elements), sphenoidal and occipital complexes. Besides a modular description of the whole skull, osteological correlates identified on the basicranium are used to infer some soft-tissue elements, especially those associated with vessels that supply the head, mainly intracranially. One of the most informative elements was the petrosal bone, whose general morphology matches that expected for a toxodont. The endocranial surface, together with the surrounding parietal, basisphenoid, occipital, and squamosal, enabled us to propose the location and communication of main venous sinuses of the lateral head wall (temporal, inferior and sigmoid sinuses), whereas the tympanic aspect and the identification of a posterior carotid artery canal provided strong evidence in support of an intratympanic course of the internal carotid artery, a controversial issue among notoungulates. Regarding the arrangement of tympanic and paratympanic spaces, the preservation of the specimen allowed us to appreciate the three connected spaces that constitute a heavily pneumatized middle ear; the epitympanic sinus, the tympanic cavity itself, and the ventral expansion of the tympanic cavity through the notably inflated bullae. We hope this study stimulates further inquires and provides potentially informative data for future research involving other representatives of the order.

## Introduction

Notoungulata is one of the most diverse orders of South American native ungulates in terms of taxonomic and morphological diversity. Although their monophyly is broadly accepted, crucial issues as their closest living relatives are still matter of debate [1–4]. Recently, new phylogenetic analyses based on proteomic data were provided. They were the first to include representatives of the South American native ungulates and their results supported the new clade Panperissodactyla, constituted by *Toxodon* (Notoungulata), *Macrauchenia* (Litopterna) and extant Perissodactyla [5–6].

Notoungulates underwent a great adaptive radiation during the Cenozoic Era resulting in a wide range of forms, from small rodent-like representatives (e.g., Suborder Typotheria) to big rhino-like forms (e.g., Suborder Toxodontia) [7–10]. Although three suborders have been traditionally recognized, Cifelli [11] and Billet [12, 13] considered only Typotheria and Toxodontia *sensu lato* (including Isotemnidae and Homalodotheriidae) as monophyletic groups. On the other hand, Notioprogonia (basal taxa from Paleocene and Eocene) is probably paraphyletic [13, 14], and the Laurasian Arctostylopidae could represent the sister group to all notoungulates [15] (but see Kondrashov and Lucas [16, 17]).

Among the Toxodontia, the “Notohippidae” are medium sized forms known from the Casamayoran South American land Mammal age (SALMA) (early Eocene) to the”piso notohippidense” of the Santacrucian SALMA (early Miocene) [8–11, 18, 19] with a peak of diversity during the Deseadan SALMA. Because of its early tendency to hypsodonty, lophodont coronal pattern and broad muzzle of some genera, late Oligocene and Miocene species have been traditionally considered grazers [18, 20]. However, neither systematic nor paleobiology of the family are thoroughly known.

Since Roth [21] proposed the order Notoungulata based mainly on posterior cranial anatomy, a good deal of attention has been paid to basicranium and auditory region when describing any representative of the order [13, 22–33]. Besides its relevance from a systematic perspective, many soft tissue structures leave osseous impressions on basicranium. The exhaustive study of this region allows inferences on major cranial arteries and veins (that supply head mainly intracranially), elements of the nervous system (i.e., exit of cranial nerves), and soft tissue elements associated with the organs of hearing and balance [30, 34–41]. Unfortunately, except for the petrosal, the rest of the auditory region and basicranium are constituted of relatively delicate elements and they are generally poorly preserved.

Although *Rhynchippus equinus* Ameghino, 1897 [42] is one of the most common notohippids in the fossil record of Patagonia, there are scarce data about its cranial morphology other than the classical descriptions which date back to the early twentieth century [22, 24, 43]. Gabbert [29] provided new data on the basicranium and auditory region of the Toxodontia based on the genera *Pleurostylodon*, *Periphragnis*, *Puelia*, *Rhynchippus*, *Leontinia*, *Ancylocoelus*, *Scarrittia*, *Homalodotherium*, *Adinotherium* and *Nesodon*. However, just some genera were figured (and not *Rhynchippus*), and no internal features were mentioned for “Notohippidae” so that the basicranial and auditory region of the family remains relatively unexplored.

In this context, the exceptionally preserved specimen MPEF PV 695 and the availability of non-invasive x-ray techniques represent an excellent opportunity to contribute to our knowledge about the family. To that purpose, we provide a detailed and comprehensive description of the cranial morphology of the species, including previously unexplored elements of the basicranium and auditory region from a typical Deseadan “Notohippidae” from Patagonia, Argentina.

### Locality and Geological Setting

**Fig 1:**
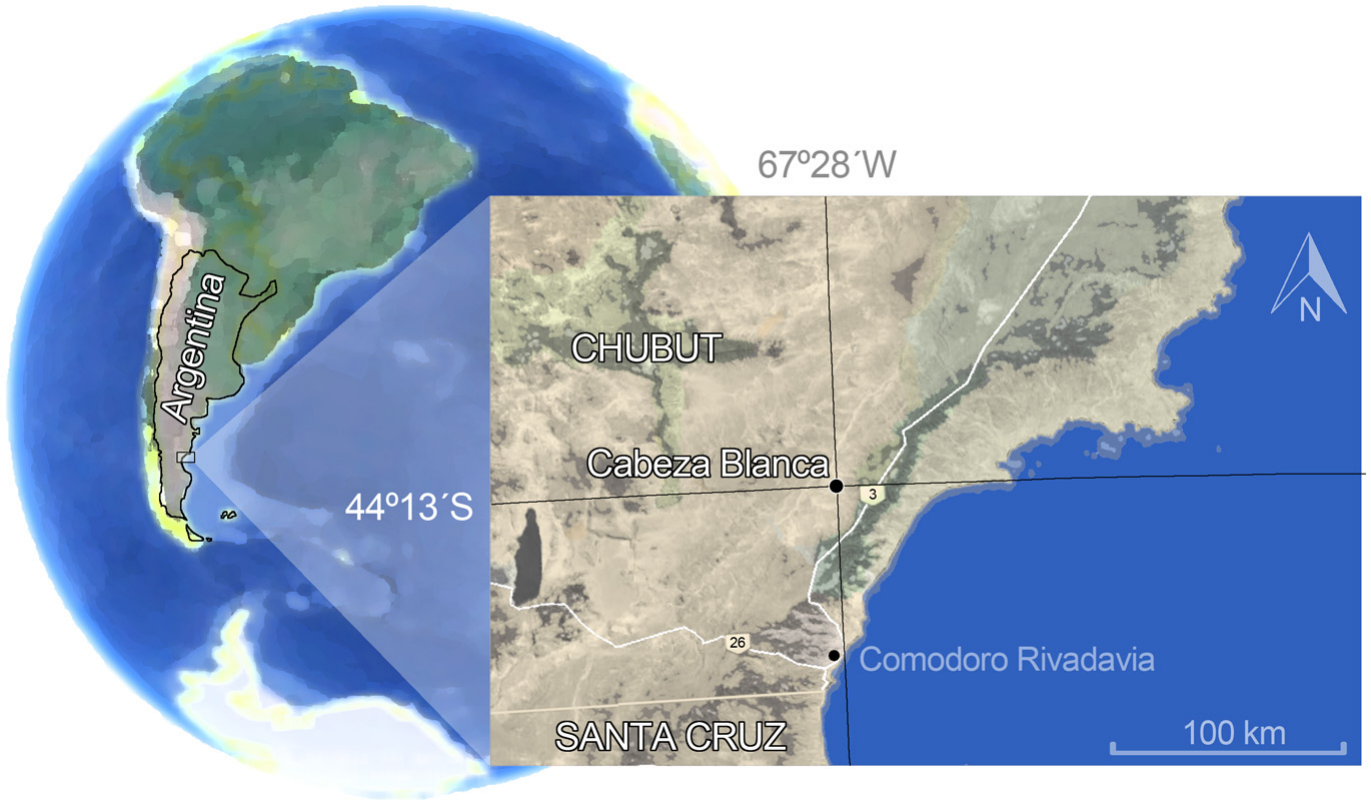
Geographic location of Cabeza Blanca locality.

The specimen MPEF PV 695 (as most of specimens of the hypodigm) was collected in Cabeza Blanca locality, SE of Chubut Province, Escalante Department, 45° 13´ S and 67° 28´ W (Fig 1). It constitutes an emblematic Deseadan locality [43–46] characterized not only by its faunal richness but also by the quality of preservation of the fossils [47, 48]. Stratigraphically, the outcrop correspond to a horizontal sequence of continental (Sarmiento Formation) and marine (Chenque Formation) sediments [49, 50]. In Patagonia, the Sarmiento Formation constitutes one of the most important and representative geological units yielding Paleogene mammals, from Casamayoran to Santacrucian SALMAs [51]. At Cabeza Blanca, two biochronological units can be clearly distinguished; the basal levels with Casamayoran fauna and the overlying levels with a typical Deseadan faunal association [50].

## Materials and Methods

The studied specimen MPEF PV 695 is a nearly complete and extremely well preserved skull. It is stored in the paleontological collection of the Museo Paleontológico Egidio Feruglio in Trelew (Chubut province, Argentina). For comparative purposes, we also examined specimens of *R*. *equinus* from the Museo Argentino de Ciencias Naturales Bernardino Rivadavia (Ciudad Autónoma de Buenos Aires, Argentina) and Field Museum of Natural History (Chicago, Illinois, U.S.A.) (Table 1). All necessary permits were obtained for the described study, which complied with all relevant regulation. Field work and fossil transportation permits were issued by the Department of Culture (Secretaría de Cultura), Government of Chubut province, Argentina. Access to private land (Estancia El Molino) was authorized by the owners. No specific permit was required for CT scanning of MPEF PV 695.

**Table 1:** Specimens examined for comparative purposes.

Specimen number	Description
MACN A 52–31	Mandible with broken ascending rami and complete dentition, fragment of left premaxilla and maxilla with left I1–M3, and isolated right M3
MACN A 52–49	Anterior portion of skull with complete dentition, except for left C (absent) and broken right I2
MACN A 52–59	Partial skull with right and left C-M3 series
FMNH P13410	Complete skull and mandible
FMNH P13420	Partial skull with complete dentition, except for right P1

For the anatomical description, and following Wible et al. [52], we divided the skull into five cranial regions: (1) nasal-facial region and cranial roof; (2) palatal region (including dental morphology); (3) orbitotemporal region; (4) basicranium and auditory region; and (5) occiput. Regarding terminology, we employed anglicized version of Latin terms following the fifth edition of the Nomina Anatomica Veterinaria (NAV). However, alternative terminology was considered when NAV was not appropriate or when more suitable or commonly used terms were available [24, 29, 33, 36, 53, 54]. To describe dental morphology, we used terminology proposed by Madden [1990, unpublished PhD dissertation] and Billet [13]. Cranial and dental measurements also follow Madden (Tables 2 and 3; S1 Fig).

**Table 2:** Cranial measurements of MPEF PV 695. Scheme of measurements provided in S1 Fig.

Measurement	Value (mm)
Total length (acrocranion to prostion) (1)	219.00
Nasal length (2)	84.30
Anterior facial width (3)	27.17
Interorbital width at the anteriormost point of the orbital rim (4)	67.30
Maximum frontal width (5)	77.14
Bizygomatic width (6)	122.50
Braincase width at the postorbital constriction (7)	37.35
I1-M3 length measured parallel to palate midline (8)	118.61
Palatal length (9)	117.10
Braincase length (10)	96.51
Muzzle width at posterior alveolar border of I3 (11)	27.92
Length of the premolar row (12)	61.69
Length of the molar row (13)	56.48
Palatal width at distal border of P1 alveoli (14)	26.09
Palatal width at distal border of P4 alveoli (15)	39.63
Palatal width at distal border of M3 alveoli (16)	69.02
Length of zygomatic arch (17)	88.33
Occipital width at the base of the paraoccipital processes (18)	68.15
Occipital height (opistion to acrocranion) (19)	44.25
Minimum supraoccipital width (20)	36.93
Maximum supraoccipital width (21)	48.84
Intercondylar width (22)	42.20
Occipital condyle width (23)	12.24
Maximum width of foramen magnum (24)	21.43
Maximum diameter of the occipital condyle (25)	20.13

**Table 3:** Dental measurements of MPEF PV 695. MD, mesiodistal diameter; EL, ectoloph length; LD, labiolingual diameter; PL, protoloph length; MDL, mesiodistal diameter measured along the lingual edge in occlusal view (Not considered by Madden [1990, unpublished PhD dissertation]); LDP, labiolingual diameter measured posteriorly, at the distal edge in occlusal view (Not considered by Madden [1990]); LCF, length of central fossette; LPCF, length of labial projection of central fossette. Scheme of measurements provided in S1 Fig.

Dental piece	Measurements (mm)							
	MD	EL	LD	PL	MDL	LDP	LCF	LPCF
Right I2	9.43	-	6.87	-	-	-	-	-
Left P1	8.00	-	9.53	-	-	-	3.33	-
Left P2	10.23	-	13.20	-	-	-	4.78	-
Right P2	10.66	-	13.14	-	-	-	5.35	-
Left P3	10.77	-	14.41	-	-	-	6.53	-
Right P3	11.11	-	14.15	-	-	-	5.85	
Left P4	14.51	-	16.25	-	-	-	8.83	-
Right P4	13.22	-	15.81	-	-	-	8.88	-
Left M1	-	19.31	-	17.76	15.12	15.10	13.92	2.78
Right M1	-	18.58	-	17.87	14.71	14.17	13.38	2.15
Left M2	-	24.87	-	18.96	16.80	11.23	13.08	2.30
Right M2	-	24.04	-	19.63	16.87	12.61	13.57	2.16
Left M3	-	19.50	-	16.35	9.38	9.99	9.87	-
Right M3	-	19.59	-	15.09	10.33	9.68	11.48	-

Besides classical methodological approach consisting of photographic record, specimen MPEF PV 695 was subjected to x-ray analysis. It was scanned at IDECH (helical mode) along the anteroposterior axis using a voltage of 120 kV and amperage of 30 mA. A total of 282 slices were obtained with a pixel spacing of 0.265 mm and interslice spacing of 0.8 mm. A second scan using a voltage of 140 kV and amperage of 60 mA was performed in order to get better resolution. It was restricted to the petrosal bone and a total of 96 slices were obtained for the target zone, with a pixel spacing of 0.195 mm and an interslice spacing of 0.5 mm. No other parameter was changed. In both cases, the image resolution was 512 x 512 pixels and raw scan data were exported from the scanner computer in DICOM format. Re-slicing of the data along the other two axes, visualization, digital segmentation and 3D reconstructions were performed using 3D Slicer v3.6.3 and 4.0.1.

### Institutional Abbreviations

AMNH VP, American Museum of Natural History, Vertebrate Paleontology, New York, U.S.A; CENPAT, Centro Nacional Patagónico, Puerto Madryn, Argentina; FMNH P, Field Museum of Natural History, Chicago, U.S.A; IDECH, Instituto de Diagnóstico del Este del Chubut, Puerto Madryn, Argentina; MACN A, Museo Argentino de Ciencias Naturales “Bernardino Rivadavia”, Ameghino Collection, Buenos Aires, Argentina; MNHN-F-BRD, Brazil fossil collection of Muséum national d’Histoire naturelle, Paris, France; MLP, Museo de La Plata, Departamento de Paleontología de Vertebrados, La Plata, Argentina; MPEF PV, Museo Paleontológico Egidio Feruglio, Paleontología de Vertebrados, Trelew, Argentina.

## Results

### Systematic Paleontology

Mammalia Linnaeus, 1758 [55]

Laurasiatheria Waddell et al., 1999 [56]

Panperissodactyla Welker et al., 2015 [5]

Notoungulata Roth, 1903 [21]

Toxodontia Owen, 1853 [57]

“Notohippidae” Ameghino, 1895 [58]

*Rhynchippus* Ameghino, 1897 [42]

#### Type species

*Rhynchippus equinus* Ameghino, 1897 [42]

#### Included taxa

*Rhynchippus equinus* Ameghino, 1897 [42]; *Rhynchippus pumilus* Ameghino, 1897 [42]; *Rhynchippus brasiliensis* Soria & Alvarenga, 1989 [59]. *Rhynchippus medianus* Ameghino, 1901 [60], is considered by Patterson (unpublished catalogue, see complete reference in [19]) a synonym of *R*. *pumilus*.

#### Geographic and stratigraphic distribution

Argentina (several Patagonian localities), Brazil (Taubate) and Bolivia (Salla). Deseadan SALMA (late Oligocene).

#### Emended diagnosis of the genus

The genus is distinguished by the following combination of characters: rounded dental arcade, similar to *Mendozahippus fierensis* and *Eurygenium pacegnum*, narrower than *Eurygenium latirostris* and clearly different from the transverse dental arcade of *Pascualihippus boliviensis*; triangular palate wider than *M*. *fierensis* and without the constriction exhibited by *P*. *boliviensis* and, to a lesser extent, by *E*. *pacegnum* and *E*. *latirostris*; two conspicuous incisive foramina on premaxillae at the anterior portion of palate; narrower and more elongate nasals compared to *Eurygenium*, with a slight constriction at the middle portion, similar to *M*. *fierensis*; robust and posteriorly directed postorbital processes; sagittal crest less developed than *E*. *latirostris* and shorter than *M*. *fierensis*; nuchal crest posteriorly projected; upper incisors decreasing in size from I1 to I3: C smaller than I3 and P1; upper premolars increasing in size from P1 to P4; high mesiolingual cingulum in upper premolars, similar to *Pascualihippus*; rhomboidal upper molars in occlusal view; central fossette opened lingually in M2-3 that eventually closes by wear in M2; convex labial face of lower incisors; well marked lingual cingulum in lower incisors, usually erased by wear; incisive-like lower canines with lingual cingulum; talonid much longer than trigonid and separated by a conspicuous labial enamel fold; meta-entoconid and ento-hypoconid folds on talonid that become fossettids and eventually disappear with wear. The diagnosis provided here is based on the original [42] and adapted to incorporate a more accurate and updated terminology.

### *Rhynchippus equinus* Ameghino, 1897

No holotype was selected from the type series in the original publication [42]. Although not formally designated, the specimen MACN A 52–31 (mandible with broken ascending rami and complete dentition, fragment of left premaxilla and maxilla with left I1–M3, and isolated right M3) was referred as lectotype by Patterson in his unpublished catalogue (see complete reference in [19]). However, since the catalogue was not published, a valid designation of a lectotype is still pending for this taxon, which is beyond the scope of this paper.

#### Referred material

MPEF PV 695, almost complete skull with left P1–M3 and right I2, P2–M3.

#### Geographic and stratigraphic provenance

The specimen MPEF PV 695 comes from Cabeza Blanca locality (Chubut Province, Argentina), middle section of Sarmiento Formation, Deseadan SALMA, late Oligocene [50].

#### Emended diagnosis of species

The species is distinguished by the following combination of characters: central fossette on upper molars anteriorly bifurcated, different from *R*. *pumilus* and *R*. *brasiliensis*, body size considerably larger than *R*. *pumilus* and *R*. *brasiliensis*; humerus with a small epicondyle; supratrochlear fossa (moderately excavated) and anconeal fossa (well excavated) connected by a conspicuous fenestra; femur with small head and short neck; pit for the rounded ligament on the posterior side of the femur head; strong and roughened greater trochanter; prominent lesser trochanter under the head of the femur; third trochanter projecting at mid-shaft level; tibia and fibula slightly longer than femur and fused on the proximal portion; tridactyl front and hind limbs.

### Description of the new specimen

The specimen MPEF PV 695 represents one of the best preserved specimens of *R*. *equinus*. The nearly complete skull belongs to an adult specimen, inferred based on the fully erupted permanent dentition and wear stage. A remarkable aspect regarding general morphology of the skull is narrower at the level of the anterior root of zygomatic arch compared to *Eurygenium* and *Pascualihippus*. This, along with the narrower muzzle, gives *R*. *equinus* a much more slender appearance when viewed dorsally.

#### Nasal-facial region and cranial roof

**Fig 2:**
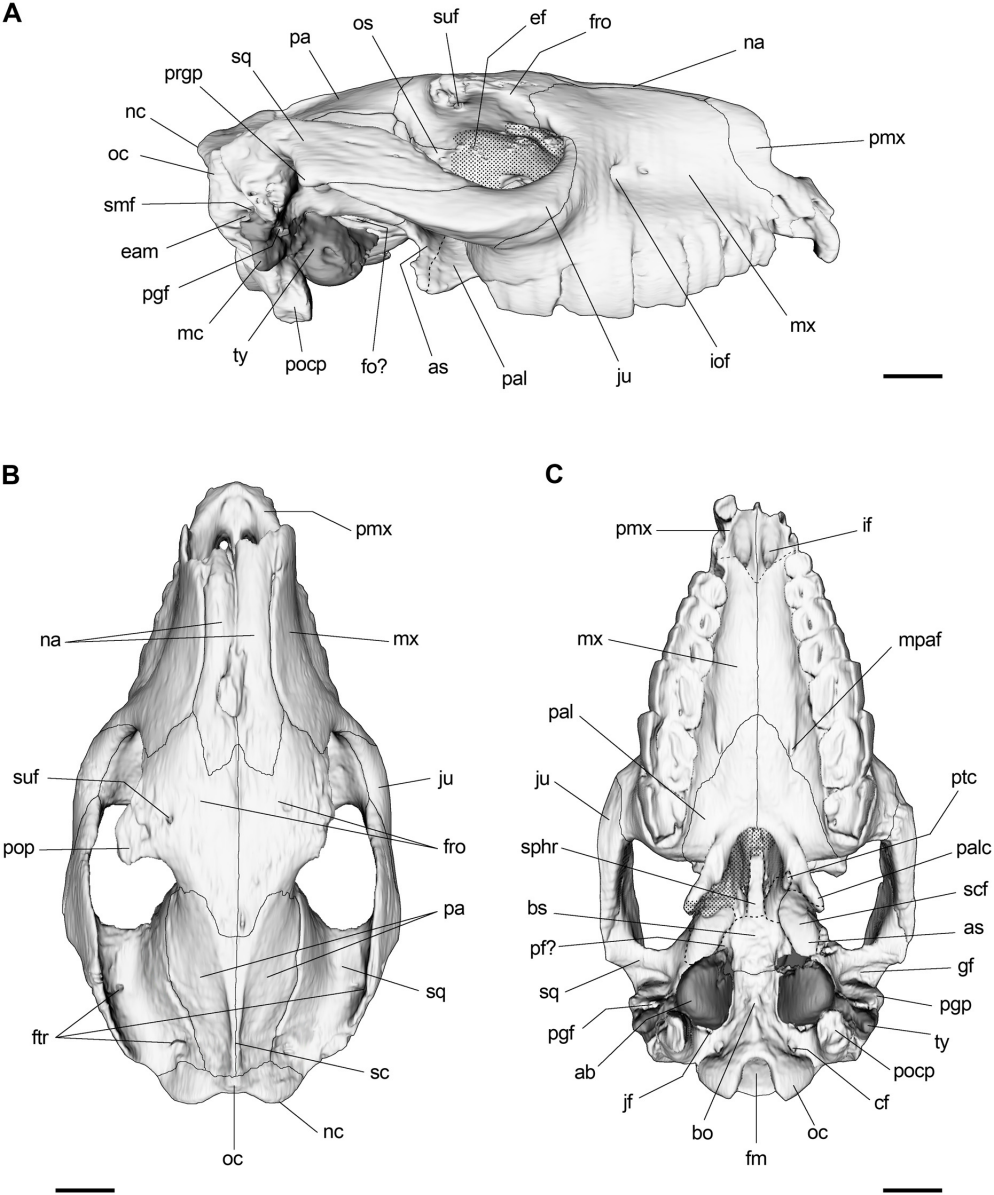
3D reconstruction of the skull of *R*. *equinus* (MPEF-PV 695). (A) Lateral view. (B) Dorsal view. (C) Ventral view. Anatomical abbreviations: ab, auditory bulla; as, alisphenoid; bo, basioccipital; bs, basisphenoid; cf, condylar foramen; eam, external auditory meatus; ef, ethmoidal foramen; fm, foramen magnum; fo, foramen ovale; fro, frontal; ftr, foramina of temporal region; gf, glenoid fossa; if, incisive foramen; iof, infraorbital foramen; jf, jugular foramen; ju, jugal; mc, meatal crest; mpaf, major palatine foramen; mx, maxilla; na, nasal; nc, nuchal crest; oc, occipital; occ, occipital condyle; os, orbitosphenoid; pa, parietal; pal, palatine; palc, palatine crest; pf, piriform fenestra; pgf, postglenoid foramen; pgp, postglenoid process; pmx, premaxilla; pocp, paraoccipital process; pop, postorbital process; prgp, preglenoid process; ptc, pterygoid crest; sc, sagittal crest; scf, scaphoid fossa; smf, suprameatal foramen; sphr, sphenoidal rostrum; sq, squamosal; styf, stylomastoid foramen; suf, supraorbital foramen; ty, tympanic. Scale bar equals 2 cm.

Premaxillae represent the anteriormost portion of the snout and constitute the base and the lateral margins of the nasal aperture. The premaxillary-maxillary suture runs from the distal part of the canine alveoli to join the lateral margin of the nasal, approximately at the level of the P3-P4 contact and assuming an S-shape course when viewed laterally. The nasals are narrow and elongate. Although they are anteriorly damaged, we can clearly appreciate that they contact the ascendant processes of the premaxillae, particularly in the better preserved right nasal. They present a slight constriction at the level of the M2 and their maximal width is registered just anteriorly to the nasofrontal suture, similar to that described for *M*. *fierensis* [61] and in contrast to the wider nasals of *E*. *latirostris*. The posteriormost extremity of the nasals extends beyond the anterior orbital rim and they are separated by the nasal processes of the frontals, giving the nasofrontal suture a W-shape, a condition also mentioned for *M*. *fierensis* [61] (Fig 2A and 2B).

The maxillae constitute much of the snout, nearly all the lateral wall of the high nasal cavity and contributes to a large portion of the secondary palate. The large and slightly oval infraorbital foramen is located at the level of the M1-2 contact and corresponds to the anterior opening of the short infraorbital canal for the passage of the infraorbital artery, vein and nerve [62]. The posterior opening of the infraorbital canal is a large and almost circular foramen at the anterior apex of the orbit below the lacrimal.

The frontals are relatively short, flat and laterally projected by the posteriorly directed postorbital processes, which are pierced by the supraorbital canal, passage of the supraorbital nerve and vessels. The ventral openings of the supraorbital canals are situated ventral to the postorbital processes on the orbital roof. About halfway between the dorsal and orbital openings, the supraorbital canals are in communication with the frontal sinuses or the diploic space. The frontal crests are just insinuated, different from the more accentuated frontal crests of *M*. *fierensis* and *E*. *latirostris*. They run posteromedially from the postorbital processes and converge to form the sagittal crest, which is less developed than in *E*. *latirostris* and shorter than in *M*. *fierensis* (Fig 2B)

The posterior cranial roof is constituted mainly by the slightly convex parietals, anteriorly separated by the posterior processes of the frontals. Posterior to the postorbital processes there is a constriction (the postorbital constriction) that coincides (intracranially) with the anterior extent of the frontal lobe [63]. This constricted portion is short (as in *M*. *fierensis*) and quite different from the more accentuated and anteroposteriorly elongated constricted portion of *E*. *latirostris*. Posteriorly, the interparietal suture runs along the sagittal crest until reaching the supraoccipital-parietal suture. Although interparietals cannot be distinguished, MacPhee [33] suggested that these elements are probably present in Notoungulata. The apparent absence of these elements in MPEF PV 695 could be due to their early fusion to surrounding elements during cranial ontogeny.

The posterior root of the squamosals contributes to the posterolateral cranial roof and constitutes the bony shell that encloses the epitympanic sinuses. A couple of vascular foramina associated with the temporal sinus can be seen at the base of the zygomatic arch and near its contact with the occipital when viewed dorsally (Fig 2B). The zygomatic process of the squamosal extends anteriorly, over the posterior part of the jugal and contributes to the laterally compressed zygomatic arch. The zygomatic arches run obliquely from the base of the orbital rim so that the glenoid fossa is located well above the orbital floor. This transversely elongated fossa is preceded by a moderately developed preglenoid process and posteriorly limited by the postglenoid (or retroarticular) process. Posteriorly, the dorsal crest of the zygomatic arches is continuous with the nuchal crest (Fig 2A and 2B).

When viewed laterally, the dorsal cranial midline is smoothly convex and the maximal height is measured at the level of the postorbital processes, in contrast to the straight dorsal profile of *M*. *fierensis* and *E*. *latirostris*. The orbits are almost circular and posteriorly opened. Ventral to the orbital rim, a moderately marked facial crest is distinguishable. The maxillary-jugal suture runs ventrally from the anteriormost point of the orbital rim and turns posteriorly accompanying the facial crest that is continuous with the ventral edge of the zygomatic arch. As already mentioned, the moderately lateral expansion of the anterior root of the zygomatic arches of *Rhynchippus* represents one of the most striking differences when compared to *Eurygenium* or *Pascualihippus*, whose anteriorly widened zygomatic arches gives those genera a more robust appearance.

#### Palatal region and dental morphology

As mentioned in the diagnosis, a “U-shaped” dental arcade is evident when viewed ventrally, in contrast to the broader dental arcade of *E*. *latirostris* and the almost straight dental arcade of *P*. *boliviensis*. The palate is triangular, different from the narrower palate of *M*. *fierensis* in which molar rows are almost parallel. Anteriorly, the most distinctive aspect of the palate is the notably large incisive foramina in comparison to *E*. *latirostris*, *P*. *boliviensis*, *E*. *pacegnum* and *M*. *fierensis* (in the last two species the incisive foramina are somewhat difficult to observe because of poor preservation of specimens in that region). They are anteriorly directed and separated by a small crest on the sagittal plane. The dorsal apertures of the incisive canals are visible on the floor of the nasal cavity. Posteriorly on the palate, the major palatine foramina (anterior openings of the posterior palatine canals) are clearly distinguishable on the maxillary-palatine suture (or even just anterior to the suture) at the level of the M2 (Fig 2C). These foramina open anteriorly into the palatine sulcus, which runs forward and parallel to the medial suture up to the level of the M1. The caudal palatine foramina (posterior opining of palatine canals) open on the orbital floor, just lateral to the medial wall into a groove so that they are not visible when viewed laterally (see below).

**Fig 3:**
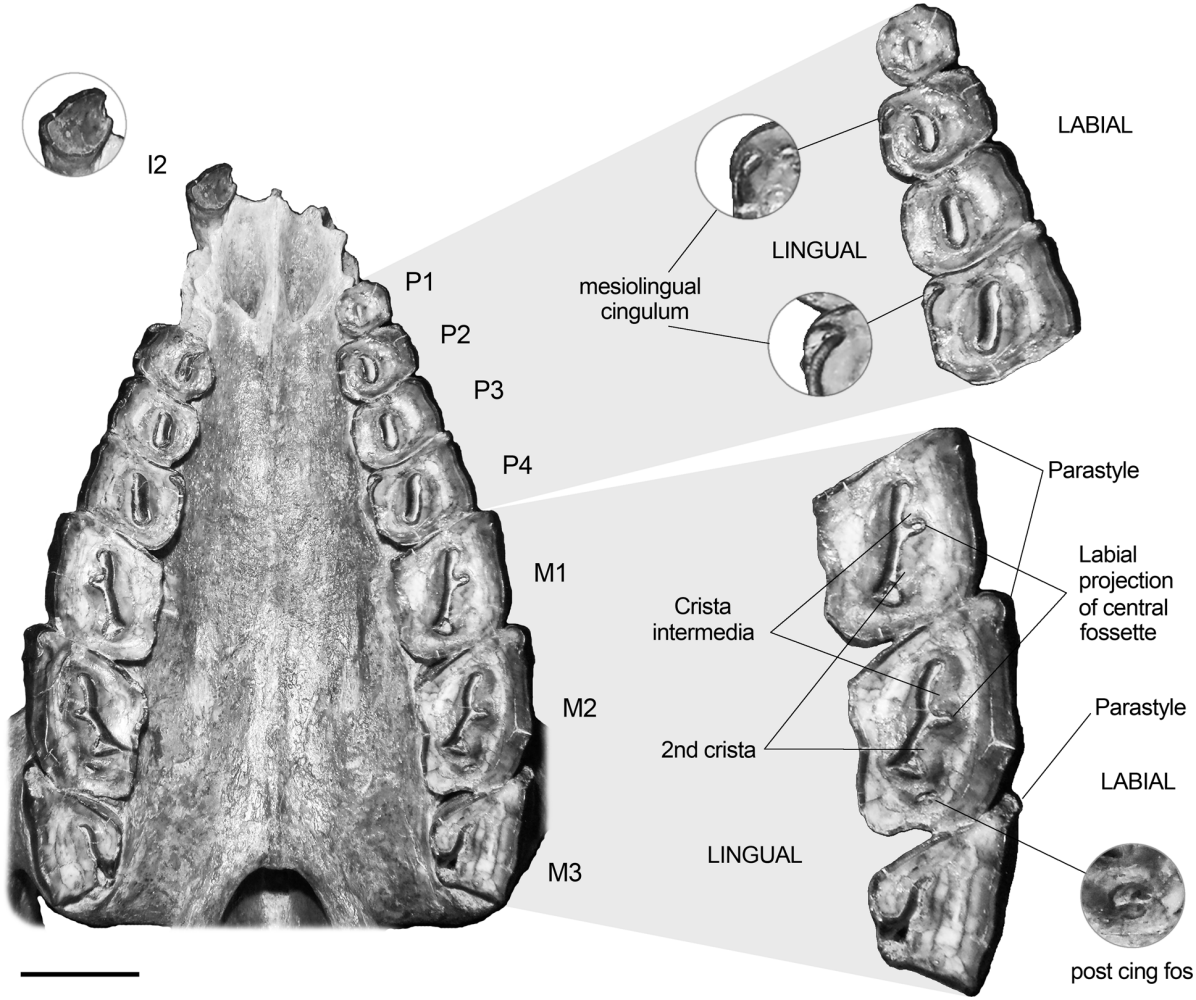
Palate and upper dentition of *R*. *equinus* (MPEF-PV 695). Scale bar equals 2 cm.

Regarding dental morphology (Fig 3), the specimen MPEF PV 695 shows a continuous tooth row (no diastema) and the typical lophodont morphology that characterizes the Toxodontia. The right I1, I3–P1 and left I1–C are not preserved in the specimen. In the original diagnosis, Ameghino [42] mentioned that *R*. *equinus* possesses a more distinctive difference in width between I1 and I3 than *R*. *pumilus*. Although neither I1 nor I3 are preserved in MPEF PV 695, we failed to recognize such difference on other specimens of the hypodigm. The only incisor (right I2) does not exhibit the longitudinal furrow mentioned by Loomis [43]. However, because occlusal morphology is usually obscured by wear, it is possible (and expectable) that the furrow had been completely erased in MPEF PV 695.

Due to their curvature, the incisors are moderately procumbent at the base but perpendicular to the plane of palate at the top. Premolars are almost quadrangular in occlusal view, except for P1 which is almost circular and notably smaller than P2. They increase in size from P1 to P4 and present a mesio-distally elongated central fossette, which runs almost parallel to the labial side of the tooth in P1-3, and obliquely in P4. The P2 and P4 show a mesiolingual cingulum. However, it is difficult to recognize a constant morphology of this feature when examining other specimens of the hypodigm. Some of them (e.g., MACN A 52–31) exhibit a mesiolingual fossette, others (e.g., MACN A 52–49) show a mesiolingual enamel infolding, and others (e.g., MACN A 52–59, FMNH P13420) present a mesiolingually opened central fossette. This variable morphology is probably related to different wear stages, as Loomis [43] mentioned when describing occlusal morphology of incisors. In the specimen described here, a mesiolingual fossette is visible on P2, no cingulum on P3, and a mesiolingual enamel infolding in P4 (Fig 3).

Molars are markedly rhomboidal in occlusal view due to the more developed paraloph, accentuated by the presence of a moderately developed parastyle. In specimen MPEF-PV 695, the central fossette of M1 and M2 shows a labial projection that divides the lingual side of the ectoloph into a crista intermedia (mesially) and a “second crista+crochet” (distally), following terminology of Billet [13]. However, this morphology seems to vary with wear, since the bifurcation of the central fossette is hardly distinguishable in specimens with more advanced wear stages (e.g., MACN A 52–31 and MACN A 52–49). Posterior to the central fossette, a small fossette (probably the postcingulum fossette) is distinguishable in the M2. The presence of this fossette also seems to be subjected to different wear stages. Similar to FMNH P13410 and different from the other specimens of the hypodigm mentioned above, the M3 is smaller than M1 and M2 because of the less development of the protoloph and a heavily reduced metaloph due to the distolingual opening of the central fossette. Although such difference seems to be explained by wear, intra-specific variability should not be discarded.

#### Orbitotemporal region

The orbitotemporal region of the specimen MPEF PV 695 is mostly well preserved, except for the anterior portion of the orbital wall, which is somewhat decayed on both sides so that sutures are not distinguishable. The lacrimal seems to be exclusively confined to the antorbital rim (there is no evidence of facial exposure) and the single, circular lacrimal foramen (not shown) is visible dorsal to the orbital aperture of the infraorbital canal when viewed posteriorly. The aforementioned suture obliteration impeded us from determining whether or not the lacrimal contacts the palatine preventing the frontal from reaching the maxilla. The ventral openings of the supraorbital canals are located high on the orbital wall just below the postorbital processes.

**Fig 4:**
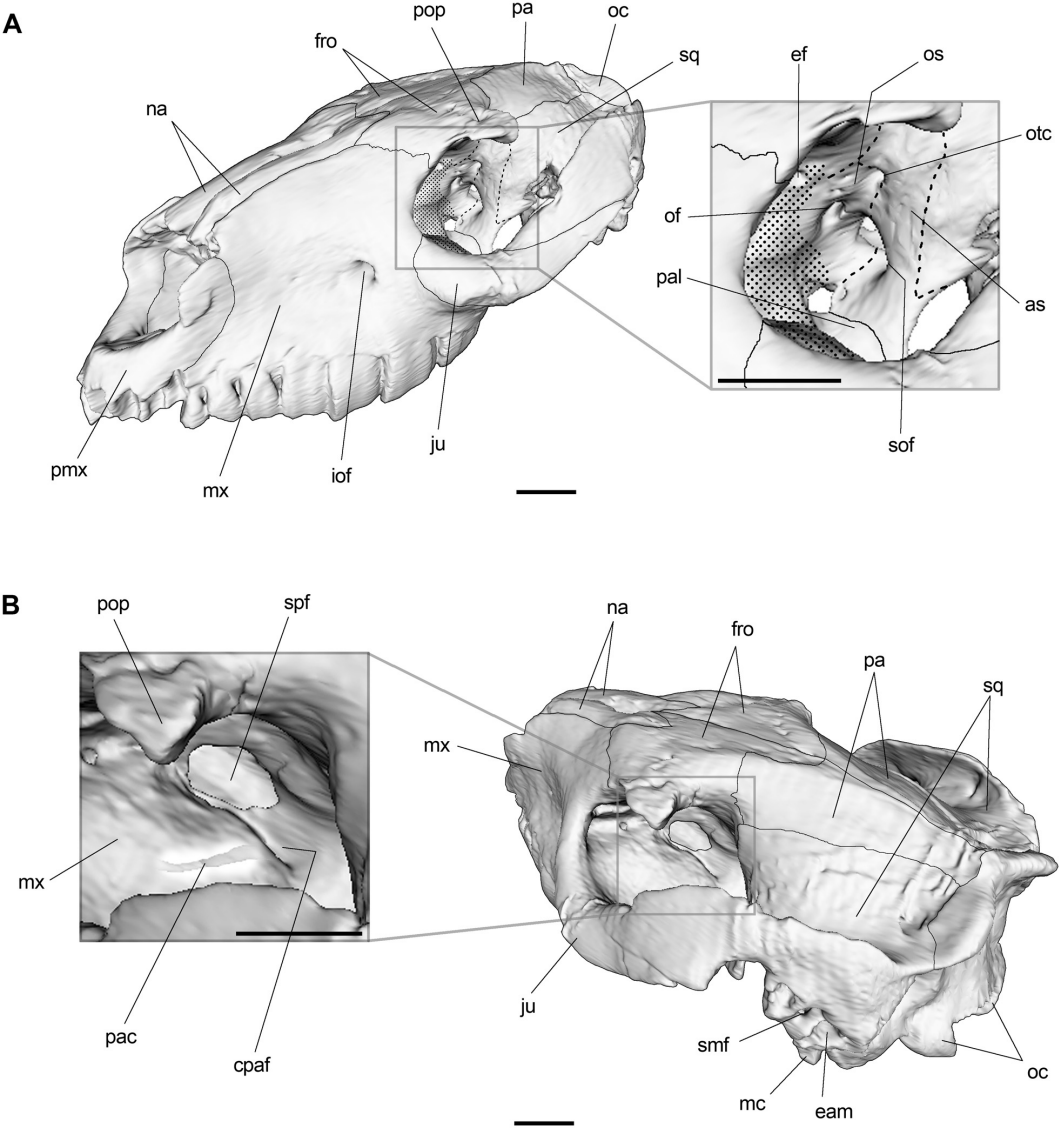
3D reconstruction of *R*. *equinus* (MPEF PV 695) and detail of the orbitotemporal region. (A) Skull oriented to show foramina on the posterior orbital wall. (B) Skull oriented to show the sphenopalatine foramen and caudal palatine foramen. Maxilla in B rendered partially semitransparent to show the palatine canal. Anatomical abbreviations: as, alisphenoid; cpaf, caudal palatine foramen; eam, external auditory meatus; ef, ethmoidal foramen; es, epitympanic sinus; fro, frontal; iof, infraorbital foramen; ju, jugal; mc, meatal crest; mx, maxilla; na, nasal; oc, occipital; of, optic foramen; os, orbitosphenoid; otc, orbitotemporal canal; pac, palatine canal; pmx, premaxilla; pop, postorbital process; smf, suprameatal foramen; sof, sphenorbital fissure; spf, sphenopalatine foramen; sq, squamosal. Scale bar equals 2 cm.

Posteroventrally, frontal contacts the dorsal margin of the rather triangular orbitosphenoid. Although the anterior extension of orbitosphenoid is difficult to appreciate, it probable reaches the posterior edge of the sphenopalatine foramen (Fig 4B). This large roughly oval opening is located anteroventrally on the orbital wall just above the posterior opening of the palatine canals, and transmits the sphenopalatine nerve, artery and vein. Posteriorly on the orbitosphenoid, the optic foramen (for the optical nerve and associated vessels) can be appreciated. Anterodorsal to the optic foramen, there is a small foramen interpreted as the ethmoidal foramen (Fig 4A).

The sphenorbital fissure is a large, oval forwardly directed opening on the back of the orbital fossa, posteroventral to the optic foramen. It probably conveys the oculomotor (III), trochlear (IV) and abducens (VI) nerves, and the ophthalmic (V1) and maxillary (V2) branches of the trigeminal (V) nerve. The orbitotemporal foramen is located dorsal to sphenorbital fissure and constitutes the orbital opening of the orbitotemporal canal, which accommodates the orbitotemporal artery and vein. These foramina are anteriorly limited by the posterior margin of the orbitosphenoid and posteriorly limited by the anterior margin of the ascending process of the alisphenoid (Fig 4A).

#### Basicranium and auditory region

The basicranium comprises the posterior cranial floor, between the choana and the occiput. Osteologically, it is formed by the posterior portion of the palatine, pterygoid and sphenoid, plus the tympanic and petrosal bone. The sphenoid (or sphenoidal complex) results from the partial or total fusion of a couple of elements that constitute an irregularly shaped bone. Except for the orbitosphenoid (which is exclusively confined to the orbital wall), the remaining elements (presphenoid, alisphenoid and basisphenoid) largely contribute to the basicranium.

**Fig 5:**
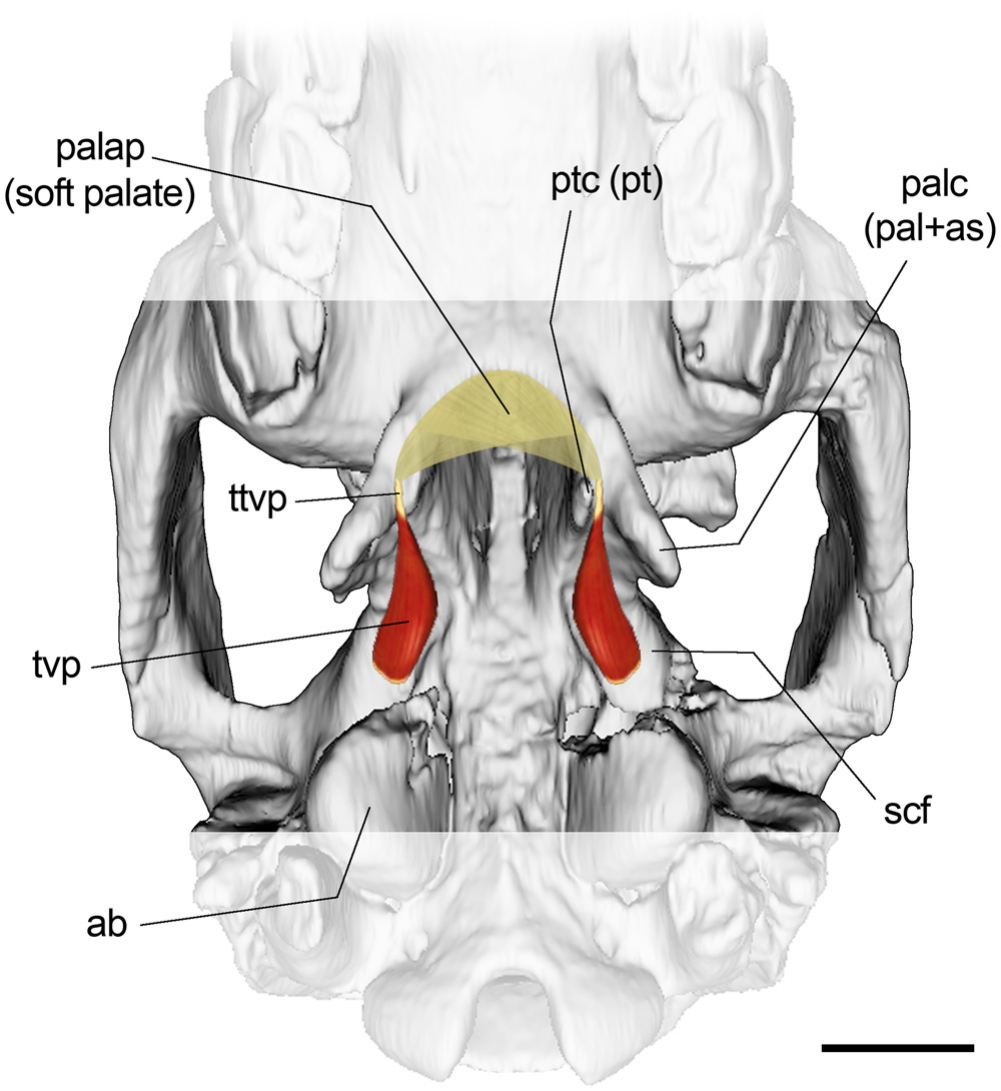
Hypothetical reconstruction of tensor veli palatini muscle and tendon in *R*. *equinus* (MPEF PV 695). Anatomical abbreviations: ab, auditory bulla; as, alisphenoid; pal, palatine; palap, palatine aponeurosis; palc, palatine crest; pt, pterygoid; ptc, pterygoid crest; scf, scaphoid fossa; ttvp, tendon of tensor veli palatini muscle; tvp, tensor veli palatini muscle. Scale bar equals 2 cm.

At the level of the choanae, the pterygoid (medial) and palatine (lateral) crests are distinguishable. Between them, and based on the specimen MLP 67-II-27-27 of *Puelia* sp., Gabbert [29] described a fossa (scaphoid fossa) interpreted as the origin of the tensor veli palatini (Fig 2C). According to her, this morphology requires the tendon of the tensor veli palatini to split the hamulus instead of passing laterally as observed in extant ungulates. However, Billet et al. [64] argues that Gabbert misidentified the pterygoid on the specimen MLP 67-II-27-27. According to him, what Gabbert identified as pterygoid was actually an extension of the palatine which, together with alisphenoid, constitutes the palatine crest. The arrangement exhibited in the specimen MPEF PV 695 supports Billet’s observation, i.e., an external crest constituted by palatine and alisphenoid, and a less developed medial crest constituted by pterygoid. Under this scenario, the tendon of the tensor veli palatini would not split the pterygoid hamulus (as argued by Gabbert). Instead, the tendon would pass laterally, between the pterygoid and palatine crests (Fig 5). This does not challenge her functional interpretation about the origin of tensor veli palatini muscle on the scaphoid fossa and the course of the tendon between the crests (regardless of the osseous origin).

Flanked by the palatine and pterygoid, the sphenoidal rostrum is exhibited as a longitudinal spine on the choanal roof when viewed ventrally. Posteriorly, the rather trapezoidal basisphenoid can be seen, laterally limited by the alisphenoids. However, the limits of these elements are barely distinguishable because of suture obliteration (Fig 2C). Endocranially, the basisphenoid exhibits a concave surface that constitutes the hypophyseal fossa of the sella turcica. The posterior margin of the basisphenoid contacts the basioccipital at level of the anterior extent of the auditory bullae.

As mentioned by Klaauw [65], the general term auditory bulla is preferred instead of the more restricted tympanic bulla (in mammals, other elements apart from tympanic bone can constitute the auditory bullae). In MPEF PV 695, the auditory bullae are well inflated so that they are clearly visible when viewed ventrally and laterally (Fig 2). They are ovoid in shape but slightly teardrop-shaped when compared to that of *M*. *fierensis*. The left bulla (anteriorly broken) allows us to observe the presence of cancellous bone tissue that thickens the wall, similar to that of other notoungulates (i.e., *Pseudotypotherium pseudopachygnathum* and *Protypotherium australe*) figured by Patterson [24] and described as a common character for toxodontians [29].

**Fig 6:**
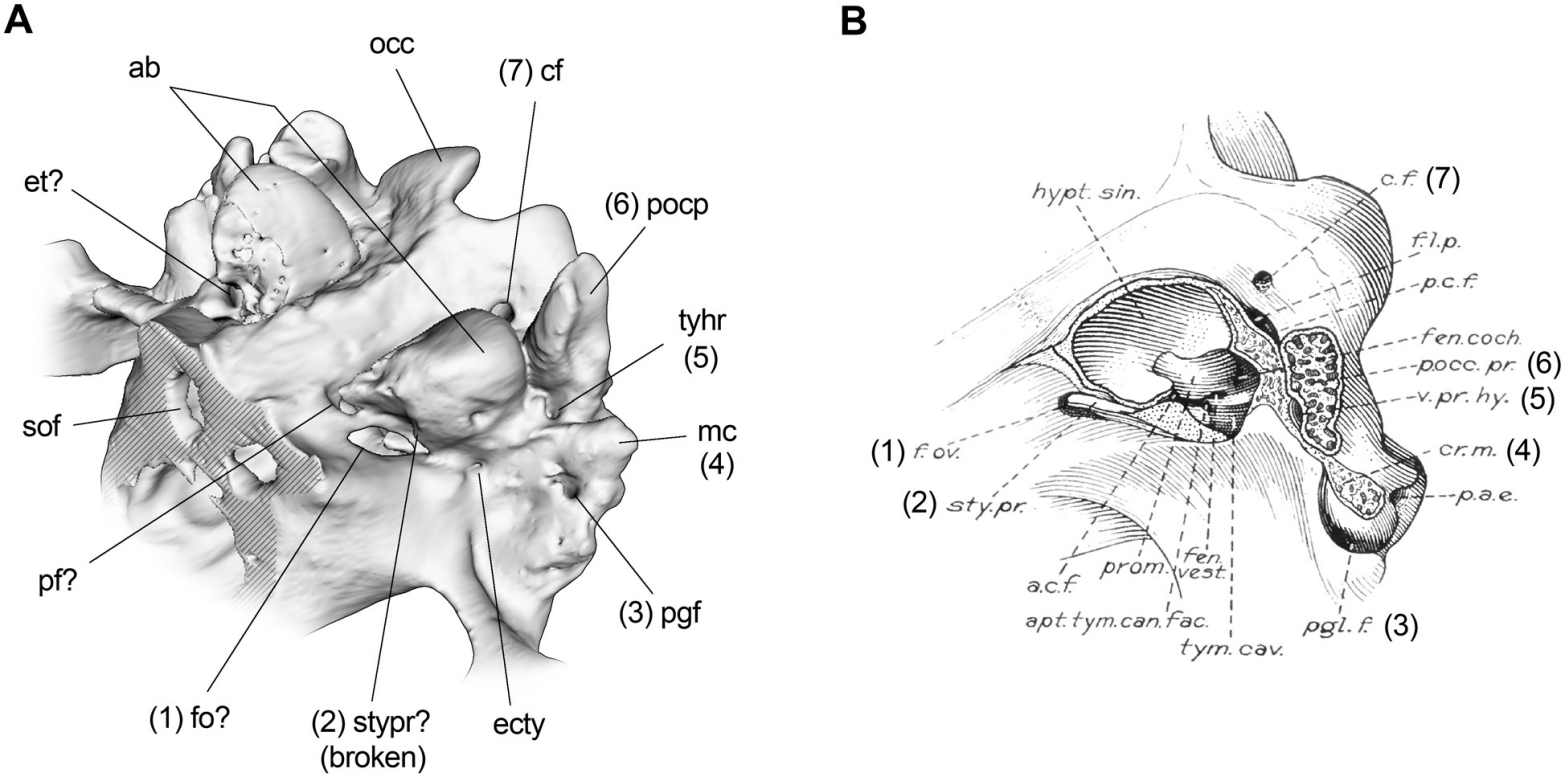
Posterior basicranial region of *R*. *equinus* (MPEF PV 695) and *Nesodon imbricatus* (FMNH P13105). (A) 3D reconstruction of *R*. *equinus*. (B) Illustration of *N*. *imbricatus* taken and adapted from Patterson [24]. Numbers in parenthesis indicate same structures in A and B. Anatomical abbreviations: ab, auditory bulla; cf, condylar foramen; ecty, exit for chorda tympani nerve; et, eustachian tube; fo?, foramen ovale?; mc, meatal crest; occ, occipital condyle; pf?, piriform fenestra?; pgf, postglenoid foramen; pocp, paraoccipital process; sof, sphenorbital fissure; stypr?, styliform process?; tyhr, tympanohial recess. Not to scale.

A barely distinguishable flange can be observed anterolaterally on the less deteriorated right bulla. This structure probably constitutes the base of the styliform process, another common feature among Toxodontia [22]. Enclosed between the styliform process and the basisphenoid, there is a conspicuous groove probably associated with the auditory (Eustachian) tube that opens dorsally into the tympanic cavity, between the tympanic and petrosal (Fig 6A). We also include in Fig 6 a restored version of an illustration taken from Patterson [24] of the specimen FMNH P13105 of *Nesodon imbricatus* (Fig 6B) in order to facilitate comparisons avoiding misinterpretations due to terminological issues. Although there is an illustration of the specimen FMNH P13410 of *R*. *equinus* [22], we chose the one of *N*. *imbricatus* because it was more detailed regarding structures that we are referring to.

**Fig 7:**
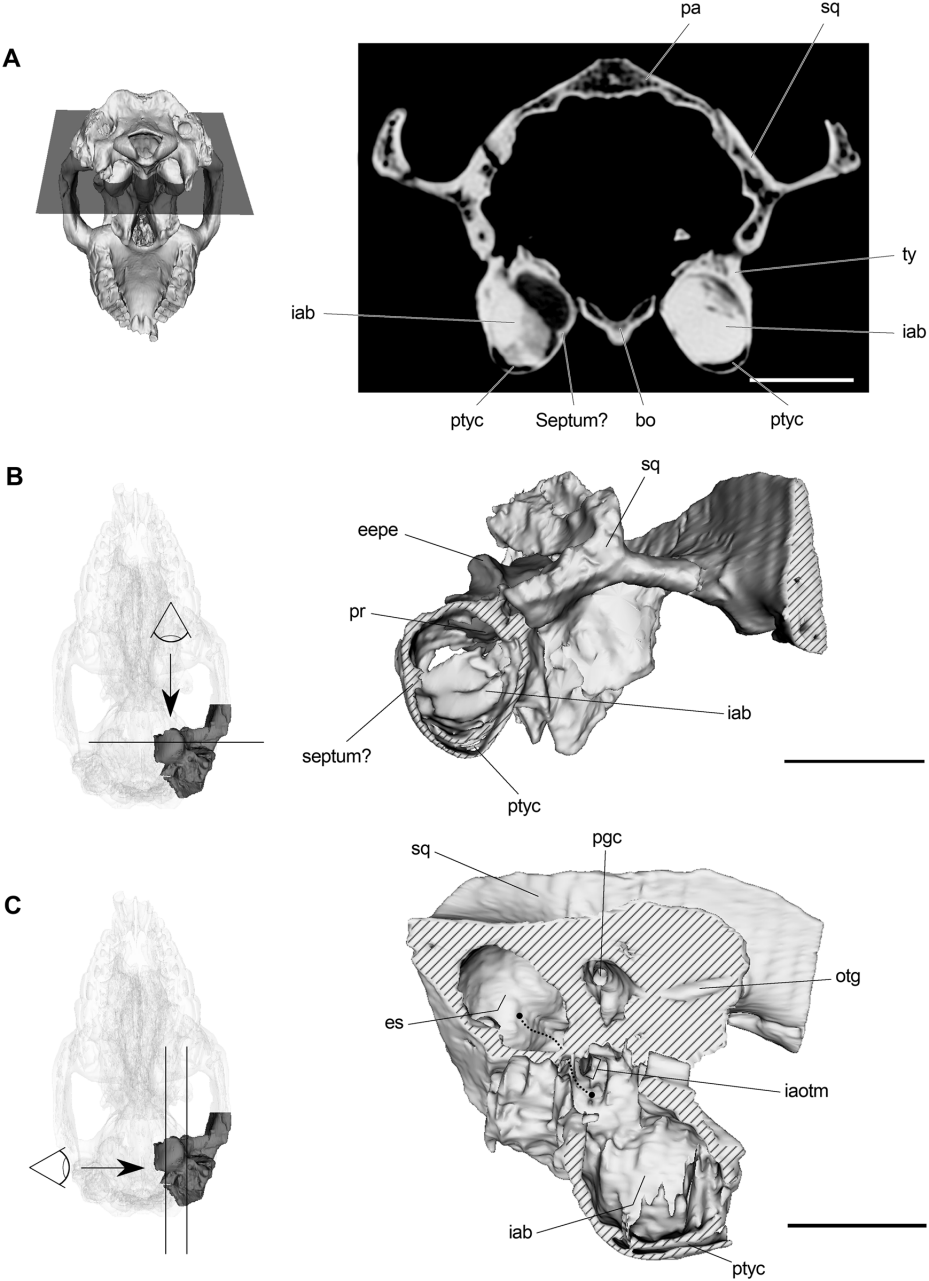
Tympanic cavity and paratympanic spaces of *R*. *equinus* (MPEF PV 695). (A) CT slice at the level of tympanic bullae. (B) Left tympanic bulla transversally sectioned to appreciate internal morphology. (C) Squamosal + left tympanic bulla parasagittally sectioned to appreciate internal morphology of tympanic cavity and epitympanic sinus. Schemas on the left indicate orientation and cut planes. Striped areas represent cut surfaces. Dotted line in C shows the connection between tympanic cavity and epitympanic sinus via the foramen pneumaticum. Anatomical abbreviations: bo, basioccipital; eepe, endocranial exposure of petrosal; es, epitympanic sinus; iab, interior of auditory bulla; iaotm, internal aperture of the ossified tubular meatus; otg, orbitotemporal groove; pa, parietal; pgc, postglenoid canal; pr, promontory of petrosal; ptyc, paratympanic cavity; sq, squamosal. Scale bar equals 2 cm.

Interiorly, CT slices show that both auditory bullae are filled with sediment (Fig 7A). However, digital removal of sediment allowed us to recognize some internal structures. A crest (vestiges of a septum?) that runs anteroposteriorlly along the medial wall of the left auditory bulla can be observed (Fig 7A and 7B). That condition could not be verified in the right bulla because it is completely filled with sediment and no internal structures are distinguishable. The presence of a septum partially dividing the tympanic cavity among notoungulates (and some considerations regarding its composition) will be discussed bellow. Ventrally on the posterior region of both bullae, a kind of “flattened” cavity can be observed (Fig 7), although its connection with the middle ear cavity cannot be distinguished.

Dorsally on the tympanic cavity, the aperture of the ossified tubular auditory meatus is hardly visible on the 3D reconstruction (Fig 7C). It seems to be located dorsolaterally, just below the pneumatic foramen that connects the tympanic cavity with the epitympanic sinuses. Despite the lack of detail on that region because of presence of sediment, the ossified tubular auditory meatus does not seem to protrude into the tympanic cavity. Epitympanic sinuses are not rare among mammals and are particularly developed in Notoungulata, associated with a significant pneumatization of the middle ear [21].

In MPEF PV 695, the epitympanic sinuses are subspherical and their size and location determine the posterior expanded morphology of the squamosals, the only elements that constitute the epitympanic theca (term proposed by MacPhee [33] to refer to the bony covering of epitympanic sinus). As mentioned above, epitympanic sinuses are anteroventrally connected to the epitympanic recess via a pneumatic foramen (aditus) on the roof of the tympanic cavity, dorsal to the internal aperture of the ossified tubular auditory meatus [24, 29].

Externally, a series of foramina can be seen on the periphery of the bullae. Although some of them are not strictly associated with an auditory function, they are located on the tympanic bone (or surrounding elements) and we consider appropriate to describe them here. Posterior to the scaphoid fossa and anterior to the auditory bullae, the exit of the mandibular branch of the trigeminal nerve (V3), nerves of the pterygoid canal and any derivative of the internal carotid artery, are observed (“sphenotympanic fissure” *sensu* Gabbert [29, 66]). Although first morphological interpretation of this fissure based on the endocast of MPEF PV 695 [63] was in agreement with Gabbert [29], a reexamination of the specimen has raised some doubts concerning its morphology. After a closer inspection, the fissure seems to be divided by a delicate strip of bone into an anterior fenestra and a posterodorsal fissure (unfortunately, this portion is broken on the left side of the skull).

Following a positional criterion, we tentatively identified the anterior fenestra as the piriform fenestra and the posterodorsal opening as the foramen ovale (Figs 2A and 6A). A similar condition was recently described by Cerdeño and Vera [67] for the Leontiniidae *Gualta cuyana*, in which the authors recognize the presence of the foramen lacerum medium and foramen oval, instead of the sphenotympanic fissure mentioned by Gabbert [29] for Toxodontia. However, we are unable to determine accurately the identity of those foramina until better preserved Toxodontia and comparative anatomical descriptions provide evidence for stronger interpretations. Posterodorsally, a small foramen that we tentatively associate to the exit of the chorda tympani, can be distinguished (Fig 6).

**Fig 8:**
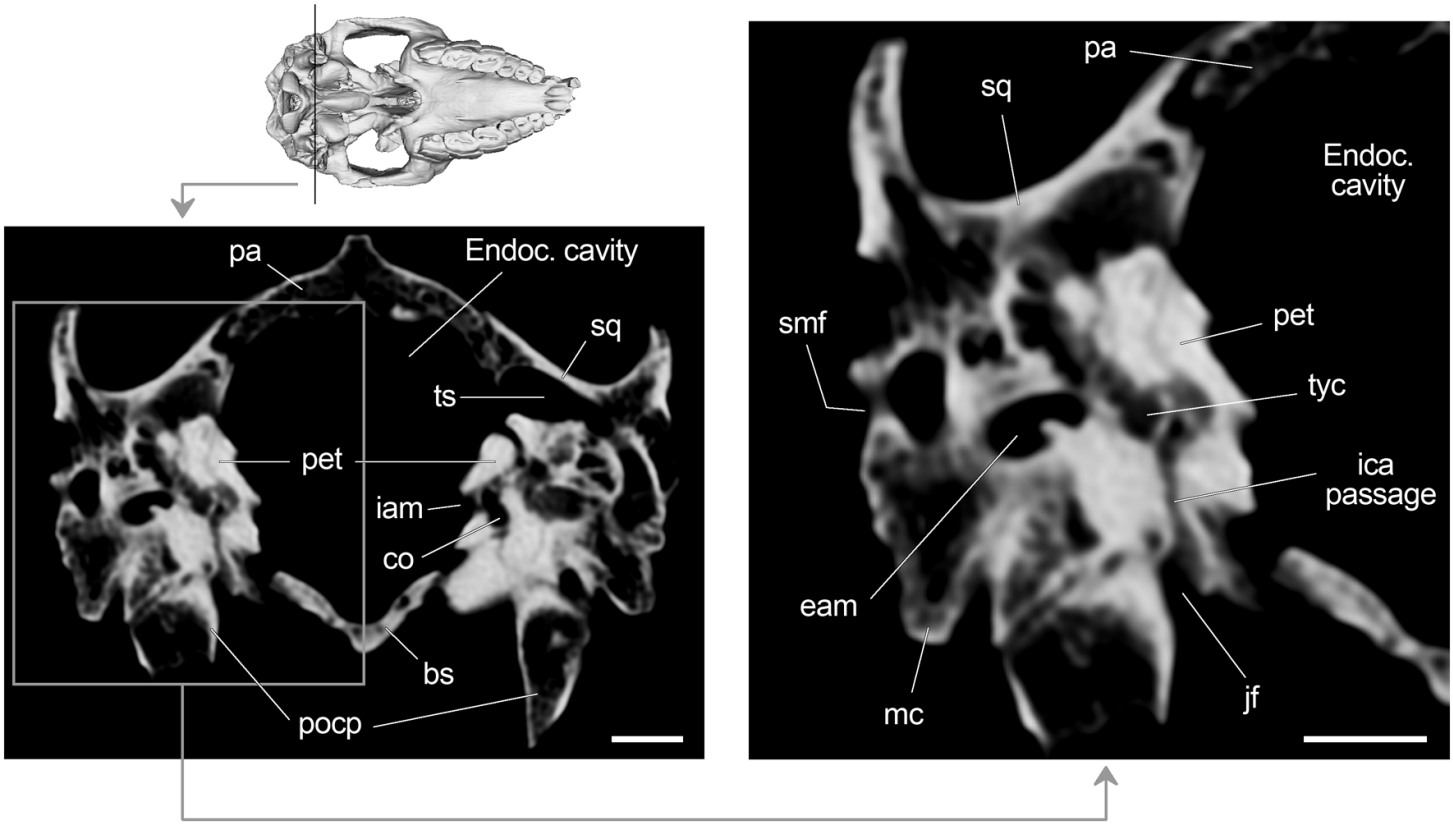
Axial CT slice of *R*. *equinus* (MPEF PV 695) at level of the jugular foramen. Anatomical abbreviations: bs, basisphenoid; eam, external auditory meatus; ica, internal carotid artery; jf, jugular foramen; mc, meatal crest; pa, parietal; pet, petrosal; pocp, paraoccipital process; smf, suprameatal foramen; sq, squamosal; ts, temporal sinus; tyc, tympanic cavity. Scale bar equals 1 cm.

The jugular foramen is visible posterior to the auditory bulla, anteromedial to the paraoccipital process and anterolateral to the condylar foramen (Fig 2C). This transversally elongated foramen transmitted the glossopharyngeal (IX), vagus (X) and accessory (XI) nerves, and sigmoid and inferior petrosal sinuses that join to form the internal jugular vein. A carefully examination of slices reveals the presence of a duct that connects the extracranial space (at the level of the jugular foramen) with the tympanic cavity (Fig 8). This canal could be for the passage of the internal carotid artery (ICA), as mentioned by Patterson [24] for Toxodontia (see discussion section).

**Fig 9:**
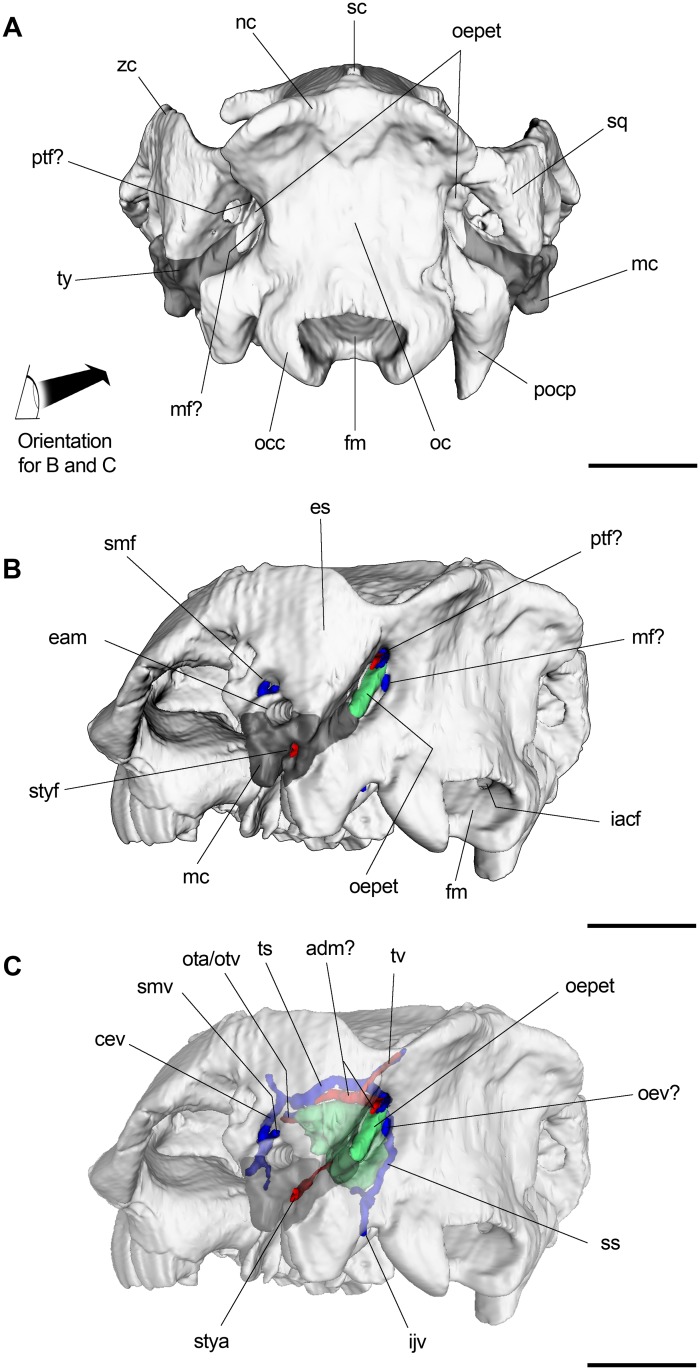
3D reconstruction of posterior skull of *R*. *equinus* (MPEF PV 695). (A) Occipital view. (B) Posterolateral view with some vascular elements reconstructed. (C) Posterolateral view with some vascular elements reconstructed (semitransparent rendered). Arteries reconstructed in red and veins (and venous sinuses) in blue. Anatomical abbreviations: adm, arteria diploëtica magna; cev, capsuloparietal emissary vein; eam, external auditory meatus; ejv, external jugular vein; es, epitympanic sinus; fm, foramen magnum; iacf, internal aperture of condylar foramen; ijv, internal jugular vein; mc, meatal crest; mf, mastoid foramen; nc, nuchal crest; oc, occipital; occ, occipital condyle; oepet, occipital exposure of petrosal; oev, occipital emissary vein; ota, orbitotemporal artery; otv, orbitotemporal vein; pocp, paraoccipital process; ptf, posttemporal foramen; sc, sagittal crest; smf, suprameatal foramen; smv, suprameatal vein; sq, squamosal; ss, sigmoid sinus; stya, stylomastoid artery; styf, stylomastoid foramen; ts, temporal sinus; tv, temporal vessels; ty, tympanic; zc, zygomatic crest. Scale bar equals 2 cm.

On the posterior face of the postglenoid (or retroarticular) process, the suprameatal foramen (oval in cross section) is clearly visible (Fig 9). This foramen conveys the suprameatal vein that receives branches that drain the temporal region (through the temporal foramina) and vessels associated with the temporal (superior petrosal) sinus. The suprameatal canal is ventrally connected to the postglenoid canal that opens posteriorly to the glenoid fossa (Fig 6) and conveys the capsuloparietal emissary vein (Fig 9B and 9C).

The external auditory meatus (circular in cross section and posterolateraly oriented) can be seen posteriorly and slightly ventral to the suprameatal foramen. It is anteroventrally limited by the meatal crest (tympanic) and posterodorsally by the squamosal. The meatal crest can be more precisely described as a bulge originating from the anteroventral margin of the external auditory meatus instead of a crest strictly defined. Posteriorly, the stylomastoid foramen is clearly visible between the posterior margin of the meatal crest and the anterior margin of the posttympanic process (Fig 9B and 9C). It conveys the facial nerve (VII) and probably the stylomastoid artery, an anastomosis (frequently observed among mammals) that irrigates the stapedial muscle, the posterior region of tympanic cavity and sometimes part of the mastoid portion, regions that earlier in ontogeny are irrigated by the stapedial artery [36].

Finally, and despite not being a foramen, it is appropriate to mention in this section the shape and location of the tympanohyal recess, defined by Billet [13] as “the fossa housing the insertion of the hyoid apparatus on the cranium of notoungulates”. It is visible as an almost circular and deep pit between the auditory bulla (anteromedially), the paraoccipital process (posteriorly) and the posttympanic process (posterolaterally) (Fig 6).

**Fig 10:**
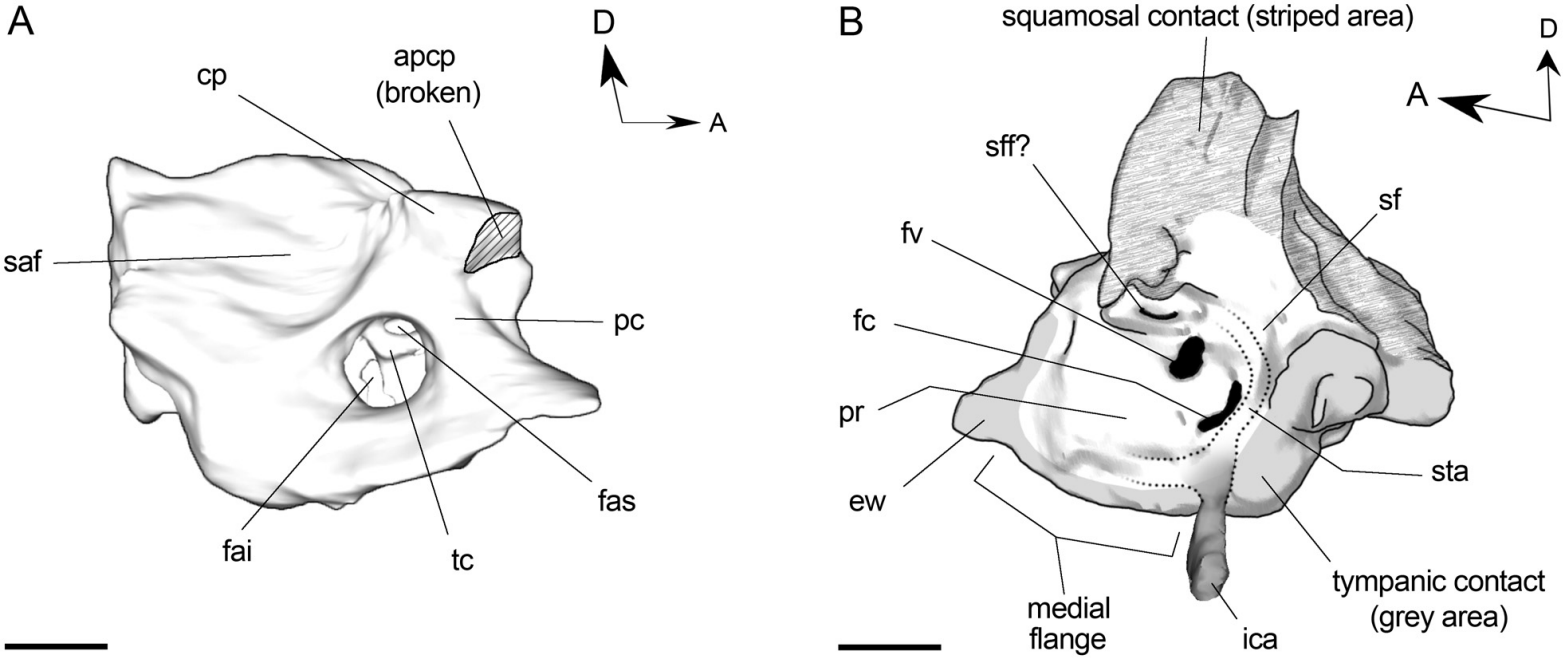
3D reconstruction of left petrosal of *R*. *equinus* (MPEF PV 695). (A) Endocranial view. (B) Tympanic view. ICA partially reconstructed and hypothetical course of proximal stapedial artery depicted. Anatomical abbreviations: apcp, anteromedial process of crista petrosa; cp, crista petrosa; ew, epitympanic wing; fai, foramen acusticum inferius; fas, foramen acusticum superius; fc, fenestra cochleae; fv; fenestra vestibuli; ica, internal carotid artery; pc, prefacial commissure; pr, promontory of petrosal; saf, subarcuate fossa; sf, stapedial fossa; sff, secondary facial foramen; sta, stapedial artery course; tc, transverse crest. Scale bar equals 0.5 cm.

Dorsal to the tympanic bone and mostly exposed endocranially, the petrosal bone constitutes the roof of the tympanic cavity. In *R*. *equinus* (and other notoungulates) this element projects posteriorly so that it can be seen on the occiput between the squamosal and occipital (see below). When viewed dorsomedially (Fig 10A) (i.e., endocranial aspect), one of the most striking features is the well developed process originating from the crista petrosa, anterodorsal to the internal auditory meatus (IAM). Although the crista petrosa is usually enlarged in notoungulates [33], not all of them possess such a projection. The process extends ventromedially forming a ledge (probably associated with a tentorial ossification) similar to that described by Gabbert [29] for *Adinotherium ovinum*. The base of the anteromedial process of the crista petrosa is continuous with the prefacial commissure (ventrally) and the crest that delimitates the anterodorsal margin of the subarcuate fossa (dorsally). The fossa (that houses the paraflocculus of the cerebellum) is wide, shallow but well demarcated. Ventrally, it is separated from the IAM by a sharp crest that runs posteriorly from the base of the ventromedial process of the crista petrosa.

The slightly posteriorly oriented IAM is almost circular in cross section and communicates the endocranial cavity with the inner and middle ear. The foramen acusticum superius (dorsal) and foramen acusticum inferius (ventral) can be identified. The former conveys the facial nerve (VII) and the latter conveys the vestibulocochlear nerve (VIII). They are separated by the transverse crest, which is distinguishable well deep in the IAM.

The ventrolateral surface (Fig 10B) (i.e., tympanic aspect) constitutes the roof of the tympanic cavity and cannot be observed in situ without removing (mechanically or virtually) the tympanic bone and part of the squamosal. The promontory (the portion of the petrosal that encloses the cochlea) is well inflated (clearly differentiable from surrounding surfaces) and oval in outline. The medial flange along with the epitympanic wing constitutes the contact to the underlying tympanic (bulla). On the posterior portion of the promontory, the fenestra cochleae (for the secondary tympanic membrane) is distinguishable. Laterally, the fenestra vestibuli (associated with the stapes) can be identified, although its shape and size is probably approximated because of the resolution of the slices.

Posteriorly, between the pars cochlearis and pars canalicularis, there is a sulcus that surrounds the promontory, probably for the passage of the facial nerve. The sulcus is continuous with the stapedial fossa (at the level of the fenestra vestibuli) and the postpromontorial fossa (posteriorly to the fenestra cochleae). Anteriorly on this trough, there is a foramen that could represent the secondary facial foramen (exit of facial nerve). Inside tympanic cavity, the chorda tympani, the stapedial nerve and the greater petrosal nerve branch off the facial nerve [68]. The greater petrosal nerve exits the tympanic cavity via the hiatus fallopii, a foramen that is generally located anteriorly on the petrosal. Unfortunately, the petrosal is somewhat decayed on that region so that the hiatus fallopii cannot be appreciated.

Finally, an interesting feature should be mentioned regarding caudal part of pars canalicularis. When examining axial CT slices at this level, a trabeculated space can be appreciated that contrast with the typically dense constitution of petrosal bone. A similar condition was mentioned by MacPhee [33] for *Cochilius volvens* (AMNH VP 29615), probably associated to the presence of hematopoeic tissue.

#### Occiput

The occiput is almost entirely constituted by elements of the occipital complex (strongly fussed basioccipital, exoccipitals and supraoccipital), plus the posterior portion of squamosals (particularly expanded in notoungulates due to the aforementioned epitympanic sinuses), the occipital exposure of the petrosal bone and posterior extent of tympanic. It constitutes the osseous elements surrounding the foramen magnum, the paraoccipital processes, the occipital condyles, the posterior wall of the braincase and the nuchal crest.

The foramen magnum is subquadrangular when viewed posteriorly, and its ventral margin is projected by a deep intercondylar notch when viewed ventrally. Anterior to the foramen magnum, the basioccipital is characterized by a marked median keel that becomes less pronounced until it disappears at the level of the contact with the basisphenoid. The occipital condyles are oval in shape with the major axis dorsolaterally oriented, so that the cranial counterparts of the atlanto-occipital joint are oblique. The conspicuous hypoglossal (or condylar) foramina, exit of hypoglossal (XII) cranial nerve, are rounded in cross section and located at the base of the occipital condyles (Figs 2C and 9B). The paraoccipital processes are located posterolaterally to the bullae and anterolaterally to the occipital condyles. Both are broken and their distal portions are lacking (Fig 2B and 2C).

When viewed posteriorly (Fig 9A), the occipitals are laterally limited by posteriormost extent of tympanics and the occipital exposure of petrosal as a strip of bone between the squamosal and occipital. Although Gabbert [29] argues that there is no occipital exposure of this element in the Toxodontia, Billet [13] coded it positively on most notoungulates in his phylogenetic analysis, including *Puelia* and *Rhynchippus*. This arrangement delimitates in MPEF PV 695 two dorsoventrally elongated foramina in each side of skull at the level of the occipital constriction (Fig 9A and 9B). One of them (mastoid foramen?) is located between the occipital exposure of the petrosal and exoccipital and the other (posttemporal foramen?) between the petrosal and squamosal. We will resume this issue when dealing with some vascular inferences in the discussion section.

Dorsally (Fig 2B), the supraoccipital contacts the posterior margins of parietals (or interparietals) just anterior to the nuchal crest. When viewed dorsally, the nuchal crest is projected backward by two lobes that clearly extend beyond the inion, similar but more accentuated than in *M*. *fierensis*. Laterally, the nuchal crest becomes less pronounced and is continuous with the dorsal crest of zygomatic arches.

## Discussion

The specimen MPEF PV 695 allowed us to notably improve our knowledge about *R*. *equinus*. Its extraordinary preservation, preparation and CT imagery gave us the opportunity not only to thoroughly describe previously unexplored characteristics of the basicranium and the auditory region, but also to deal with some relevant aspects regarding cranial circulation based on its osteological correlates.

### Auditory bulla

The auditory bulla of notoungulates has always been a matter of debate, which is natural considering the relevance of the auditory region for the systematic of the order. Patterson [24] proposed a compound bulla based on the presence of a septum that could indicate the contact between ectotympanic and entotympanic bones. Among the Typotheria, he mentioned the presence of a vertical septum in *Hegetotherium*, *Pachyrukhos* and *Pseudotypotherium*, and “vestiges” of a septum in *Interatherium* and *Protypotherium*. Among the Toxodontia, he mentioned the presence of a horizontal septum in *Nesodon*, *Adinotherium*, *Rhynchippus* and *Ancylocoelus*. Although he latter reinterpreted his own observations regarding a compound bullae in notoungulates [69], he continued to consider it likely.

However, as argued by MacPhee [33], the presence of a septum is not enough to evaluate whether or not the auditory bulla is composed of more than one element, especially in those groups with a significant middle ear pneumatization. Among the numerous notoungulates he examined, he only found evidence of a compound bulla in *Cochilius volvens*, based on a partially obliterated suture that could indicate the contact between ento and ectotympanic. Unfortunately, he focused on interatheriids and hegetotheriids (Typotheria), precluding close comparisons to “Notohippidae”. The most recent contribution dealing with this issue in Toxodontia is that of Gabbert [29], who failed in identifying any suture on the external bullar wall, leaving this question unresolved.

As mentioned when describing the interior of the auditory bullae, we barely distinguished an incomplete ridge on the medial wall of the left auditory bulla (Fig 7B and 7C). In case of being a septum (and not an artifact of preservation), no evidence of a suture or constriction can be distinguished externally on the bullar wall that could be interpreted as the contact between the ecto and entotympanic. Thus, in agreement with MacPhee [33] for typotherians (except for *Cochilius*) and Gabbert [29] for toxodonts, we could not find enough evidence to corroborate the existence of a compound auditory bulla in *R*. *equinus*.

Regarding the paratympanic spaces, the epitympanic sinuses do not show any striking feature. On the other hand, the flattened paratympanic cavity on the ventral bullar wall has not been previously reported for a toxodont. The only comparable cavity mentioned for a notoungulate is a medial paratympanic cavity in *Protypotherium* sp. (MLP 12–2780), described by MacPhee [33] as an expanded paratympanic space “that inflated the entire medial bullar wall up to the point where the bulla meets the paraoccipital process…”. A detailed study of this region in *R*. *equinus* (and other toxodonts) would be highly recommended in order to determine whether or not it is homologous to the medial paratympanic cavity of *Protypotherium*.

### Comments on inferred vascular circulation

**Fig 11:**
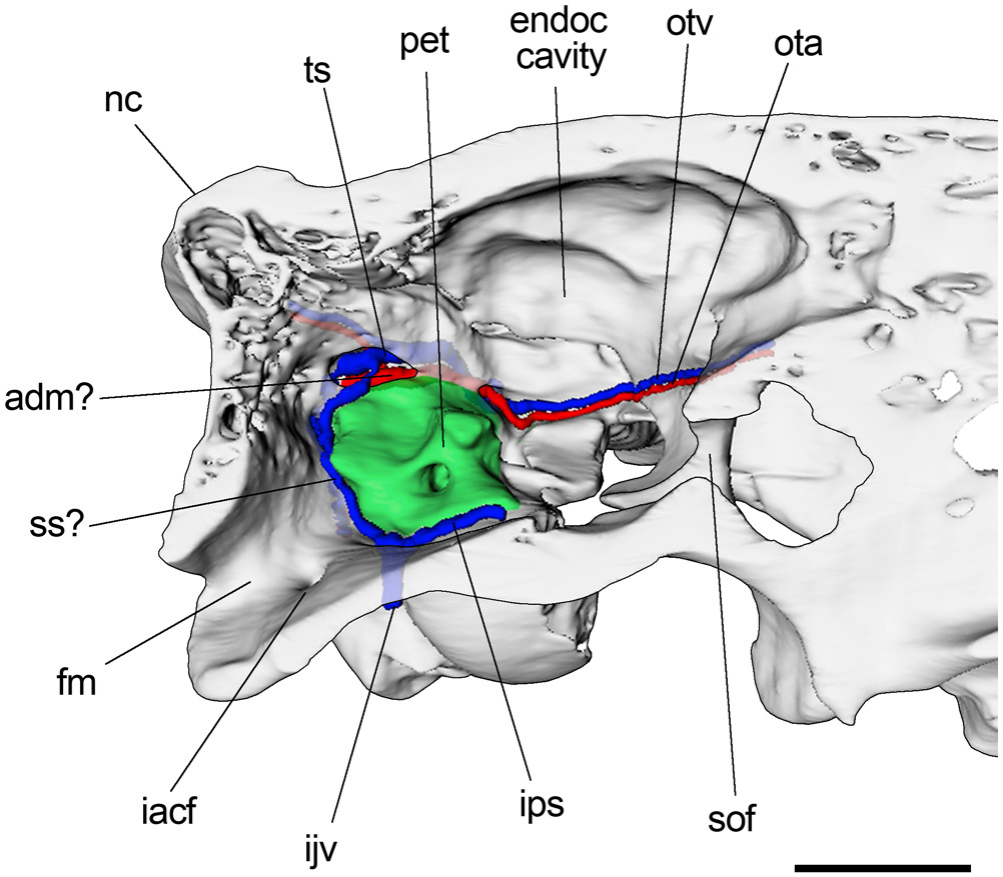
Parasagitally sectioned 3D reconstruction of posterior skull of *R*. *equinus* (MPEF PV 695). Internal surface of the braincase wall and some reconstructed vascular elements. Arteries reconstructed in red and veins (and venous sinuses) in blue. Anatomical abbreviations: adm?, arteria diploëtica magna?; cp, crista petrosa; fm, foramen magnum; iacf, internal aperture of condylar foramen; ijv, internal jugular vein; ips, inferior petrosal sinus; nc, nuchal crest; ota, orbitotemporal artery; otv, orbitotemporal vein; pet, petrosal; sof, sphenorbital fissure; ss?, sigmoid sinus?; ts, temporal sinus. Scale bar equals 2 cm.

The examination of the endocranial surfaces of braincase allowed us to observe the presence and location of some sulci, probably associated with venous sinuses and other vascular elements (Fig 11). Dorsal to the petrosal (between this bone and the squamosal), an extremely well marked sulcus can be appreciated. It probably accommodates the temporal (or petrosquamosal) sinus. The temporal sinus receives the transverse sinus (not reconstructed) and branches from the temporal region. Anteriorly, the temporal sinus drains through the capsuloparietal emissary vein, which exits the cranium via the postglenoid foramen and drains into the external jugular vein (Fig 9C). The posterior distributary branch of the transverse sinus is the sigmoid sinus [37,70]. Although we failed to find a sulcus or a canal unambiguously associated with the sigmoid sinus, it could be accommodated between the mastoid portion of the petrosal and the occipital. In placentals, the sigmoid sinus is ventrally connected to the internal jugular vein at the level of the jugular foramen [37] (Fig 11).

It is worth mentioning in this instance some considerations regarding the aforementioned foramina on the occiput. A paired foramen has been recognized when describing the occiput in a variety of notoungulates, generally referred as the mastoid foramen [21, 32]. However, since Kramarz et al. [71] argued that there was no reason to consider these foramina as the mastoid foramina instead of the posterior opening of the posttemporal canal, a good deal of attention has been paid to this issue [32, 33, 72].

As mentioned when describing the occiput, we recognized in MPEF PV 695 two paired foramina, separated by the occipital exposure of the mastoid portion of the petrosal. This was unexpected because it has not been mentioned before for other notoungulates. We tentatively identified the lateral one (between the petrosal and squamosal) as the posttemporal foramen since it opens intracranially into the sulcus for the temporal sinus. The other foramen (medial to posttemporal foramen, between the occipital and petrosal) could be the true mastoid foramen since it opens intracranially near the less marked sulcus that we associate with the sigmoid sinus (Fig 9C). In mammals, the posttemporal foramina transmit the arteria diploëtica magna and venous vessels (vena diploëtica magna?) linked to venous sinuses of the lateral braincase wall, whereas the mastoid foramen transmits an emissary vein connected intracranially to the sigmoid sinus [36, 37, 54, 73–75].

Similar to sigmoid sinus, no groove for the inferior petrosal sinus (petrobasilar canal) can be appreciated. However, the ventromedial margin of the petrosal does not contact the lateral margin of the basioccipital so that the subjacent tympanic is visible when viewed endocranially. As a result, a trough is defined between medial margin of the petrosal and the lateral margin of the basioccipital that could accommodate the inferior petrosal sinus (Fig 11). As mentioned by Billet and Muizon [32], an intracranial course of this sinus would be expected since it is considered as a synapomorphy of Placentalia [38]. In mammals, the inferior petrosal sinus is anteriorly communicated to the cavernous sinus (lateral to the hypophysis) and posteriorly confluent with the sigmoid sinus [76].

At the level of the middle ear and regarding ICA course, the aforementioned communication between extracranial and intratympanic spaces at the level of the jugular foramen is interpreted as the posterior carotid canal for the passage of the ICA (Fig 8). Patterson [24] proposed an intratympanic ICA course for the Toxodontia and some Typotheria (Interatheriidae and Mesotheriidae) based on the presence of a posterior carotid foramen which would transmit the ICA into the tympanic cavity. In contrast to Patterson, Gabbert [29] failed in identifying such a foramen in any of the specimens she described and concluded that no strong evidence was available to determine whether or not the ICA course was intratympanic.

Posteriorly, Billet et al. [64] identified a posterior carotid foramen (confluent with posterior lacerate foramen) in the Mesotheriidae *Trachytherus alloxus*, and Billet and Muizon [32] mentioned the presence of a foramen piercing the posterior wall of the bulla in the toxodontians *Pleurostylodon*, *Nesodon* and *Ponanskytherium*, and in the typotherian *Plesiotypotherium achirense*. In this context, the morphology described here for MPEF PV 695 could represent strong evidence of an intratympanic course of the ICA among the Toxodontia. The fact that ICA passage was only distinguishable on CT slices would explain why Gabbert [29] could not identify it in the taxa examined by her.

Once inside the tympanic cavity, the course of ICA is not evidenced by any groove on the promontory. Billet and Muizon [32] could not distinguish any groove on the promontory of MNHN-F-BRD 23 (an isolated petrosal referred to a basal Notoungulate indet.) and suggested this condition (absence of grooves) as a plesiomorphic trait for the order. Anteriorly on the bulla, we could not find an anterior carotid foramen for the exit of the ICA. However, as proposed by some authors [24, 29, 32], the ICA could abandon the tympanic cavity via the piriform fenestra or the anterior lacerate foramen.

Likewise, it was not possible to distinguish any groove associated with the stapedial artery, originated from the ICA at the level of the tympanic cavity. Billet and Muizon [32] proposed the existence of a stapedial system in MNHN-F-BRD 23 based on the presence of a canal on the tegmen tympani and a sulcus on the anterolateral portion of petrosal. They interpreted this morphology as the pathway of the superior ramus of the stapedial artery before joining the arteria diploëtica magna. Although that interpretation is congruent with MPEF PV 695, the lack of an appropriate resolution to such a detailed observation prevented us to confirm that pattern. In this context, we can only infer the course of the proximal stapedial artery (Fig 10).

### Systematic considerations

As mentioned when characterizing the order, the evolutionary relationship between notoungulates and living mammals is matter of debate [1–5]. The phylogenetic hypothesis provided by O´Leary et al. [3] shows the notoungulate *Thomashuxleya externa* (close related to “Notohippidae”) within Afrotheria, whereas the phylogeny obtained by Beck and Lee [4] grouped notoungulates (represented by *Henricosbornia*, *Simpsonotus* and *Thomashuxleia*) and pyrotheres (represented by *Pyrotherium*) within Laurasiatheria. The later is supported by the aforementioned phylogenetic analyses based on molecular data [5, 6], which are consistent not only with the traditional interpretation about the origin of the South American native ungulates [77] but also with the Meso-Cenozoic evolution of the paleogeographic relation between North and South America [78].

In this context, we could not corroborate most of the afrotherian cranial synapomorphies proposed by O´Leary et al. [3]. The only three “afrotherian-like” characters identified in MPEF PV 695 were the dorsal margin of external auditory meatus lower than highest point of ventral margin of zygomatic process, the contribution of the supraoccipital to the mastoid foramen, and the rounded upper incisors roots (S1 Table). Although we also fail in identifying some of the cranial synapomorphies proposed for Laurasiatheria, several characters were not applicable (or at least questionable) when examining MPEF PV 695 and could not be properly evaluated (S2 Table). Besides, considering that several synapomorphies referred to mandible and postcranium [3], a thoroughly revision of specimens in which these elements are preserved would represent a valuable complement to our observations.

Regarding the systematic of the order, some relevant aspects of both, tympanic and endocranial surfaces of the petrosal bone should be noted. The inflated promontory of *R*. *equinus* (clearly distinguishable from the surrounding surfaces) was expectable since it is a common trait in notoungulates (Fig 10B). Billet and Muizon [32] have reported the same morphology for MNHN-F-BRD 23 and proposed it as a synapomorphy of the order. Among Toxodontia, a similar morphology has been documented for *Scarrittia canquelensis* [13, 29] and *R*. *pumilus* (GM, pers. obs.).

The inflated morphology of the promontory contrasts with the relatively flattened medial flange. In *R*. *equinus*, the medial flange is less expanded than in *S*. *canquelensis* and *E*. *latirostris*, but clearly more than in MNHN-F-BRD 23. As expected for a Toxodontia, the oval outline of promontory differs from the “strongly curved bean shaped promontory” described by Billet and Muizon [32] for *Protypotherium*, *Hegetotherium* and *Plesiotypotherium*, which is considered a derived condition of Typotheria. Regarding the periphery of promontory, a well marked trough seems to be the confluence of the facial sulcus, stapedial fossa and postpromontorial fossa. This condition was also mentioned for *Scarrittia* and *Protypotherium* and was proposed as another synapomorphy for the order [32].

When viewed endocranially, (Fig 10A), the shallow subarcuate fossa is similar to that described for *Adinotherium ovinum* [29], consistent with the morphology expected for a toxodont according to Billet and Muizon [32]. As in *A*. *ovinum*, *Gualta cuyana* and *Leontinia gaudryi*, no petromastoid canal is distinguishable at the bottom of the fossa. Regarding IAM, a deep location of the crista transversa within the meatus is a characteristic also exhibited by *Adinotherium* and *Scarrittia*. This condition clearly differs from the shallow location observed in MNHN-F-BRD 23, *Hegetotherium* and *Notostylops* [32].

On the other hand, the presence of a marked crest separating the subarcuate fossa from the IAM (as observed in MPEF PV 695) seems to be phylogenetically useless since it is present in *Notostylops*, *Hegetotherium*, *Protypotherium* [32], *Colbertia lumbrerense* [79] and *L*. *gaudryi* (GM, pers. obs.), but absent in *Trachytherus* [64] and *G*. *cuyana* [67]. Although contributions mentioned above have notably increased our knowledge about petrosal bone, we are far from having a general overview of its morphological diversity among notoungulates.

## Conclusions

The extraordinary preservation, preparation and CT imagery of MPEF PV 695 allowed us to describe previously unexplored characteristics of the basicranium and auditory region of *R*. *equinus*, a typical notohippid from the Deseadan SALMA of Patagonia. Although representing partial information (neither mandible nor postcranial were evaluated), cranial morphology seems to be more consistent with the hypothesis of notoungulates belonging to Laurasiatheria, as suggested by some recent high-level phylogenies [4–6]. Within the order, the tympanic and endocranial surfaces of the petrosal bone of MPEF PV 695 are congruent with the morphology expected for a member of Toxodontia.

Regarding the auditory region, we can roughly recognize three connected spaces that constitute a heavily pneumatized middle ear: (1) the tympanic cavity itself, (2) the posterodorsal epitympanic sinus and (3) the ventral expansion of the tympanic cavity through the notably inflated bullae (hypotympanic sinus *sensu* Patterson [24]). The presence of a septum partially dividing the tympanic cavity is still tentative, and a compound bulla could not be confirmed. Ventrally on the bullar wall, a hollow space (paratympanic cavity?) is clearly distinguishable (Fig 7). Among notoungulates, MacPhee [33] mentioned the presence of a medial paratympanic cavity in *Protypotherium* sp., but further work is required to propose hypotheses of homology and/or functional comparisons between them.

When examining the endocranial surface of the lateral braincase wall, the sulci for the temporal (superior) sinus and, to a lesser extent, the sigmoid (posterior) sinus are visible on the periphery of the petrosal. Ventrally, the inferior petrosal sinus may be located between the ventromedial margin of the petrosal and the lateral margin of the basisphenoid (Fig 11). Although scarce data are available other than provided here, work in progress in other toxodonts leads us to think that this arrangement might not be exclusive of notohippids.

Concerning arterial circulation, the connection between the jugular foramen and tympanic cavity (Fig 8) is particularly relevant. This communication probably represents the posterior carotid canal and we consider it as strong evidence of an intratympanic course of the ICA among Toxodontia. Inside the tympanic cavity, the path of the ICA (and stapedial artery, if present) cannot be precisely reconstructed because of lack of grooves on the promontory, and only a hypothetical course can be suggested based on Wible [36] and Billet and Muizon [32] (Fig 10).

On the occiput, and strongly related to posterior cranial circulation, two paired foramina separated by the occipital exposure of the petrosal are recognized. Based on a positional criterion, we tentatively identified the lateral one (between petrosal and squamosal) as the posttemporal foramina, whereas the medial one (between petrosal and occipital) is identified as the true mastoid foramina (Fig 9).

Given that notoungulates are largely defined by synapomorphies related to the posterior skull, detailed descriptions of this region (facilitated by non-invasive techniques such as CT scanning) are inherently valuable. Moreover, considering the apparent “conservative arrangement” of certain structures of the basicranium, paleobiological issues (e.g., soft-tissue inference) based on well preserved specimens as MPEF PV 695 not only provide reliable data on the species but also represent potentially informative data for further research in other representatives of the order.

## Supporting Information

S1 Fig Scheme of cranial and dental measurements. Cranial and dental measurements were taken and adapted from RH Madden unpublished PhD dissertation “Miocene Toxodontidae (Notoungulate, Mammalia) from Colombia, Ecuador and Chile” (Duke University). See Tables 2 and 3 for references.
(TIF)

S1 Table Afrotherian cranial synapomorphies and condition observed in MPEF PV 695. Afrotherian cranial synapomorphies listed by O’Leary et al. [3] and condition observed in *R*. *equinus* based exclusively on specimen MPEF PV 695. Numbers in parenthesis indicate character number in the original analysis.
(PDF)

S2 Table Laurasiatherian cranial synapomorphies and condition observed in MPEF PV 695. Laurasiatherian cranial synapomorphies listed by O’Leary et al. [3] and condition observed in *R*. *equinus* based exclusively on specimen MPEF PV 695. Numbers in parenthesis indicate character number in the original analysis.
(PDF)
