# Supplementary figures and images for: Cranial Morphology of the Late Oligocene Patagonian Notohippid Rhynchippus equinus Ameghino, 1897 (Mammalia, Notoungulata) with Emphases in Basicranial and Auditory Region
Source: PLoS One. 2016 May 27;11(5):e0156558. doi: 10.1371/journal.pone.0156558 (PMC4883762; doi:10.1371/journal.pone.0156558)

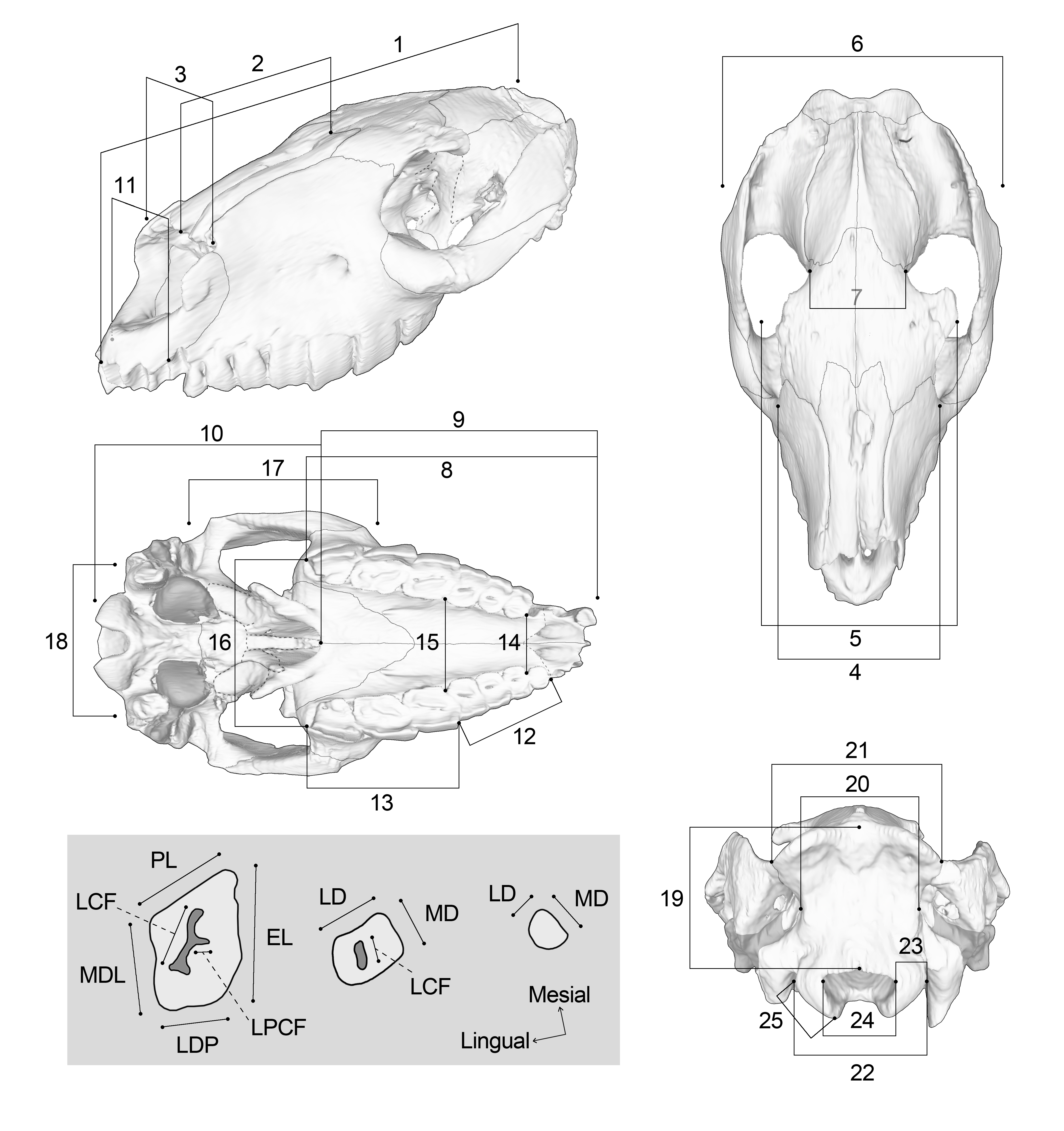

Supplement: S1 Fig — Cranial and dental measurements were taken and adapted from RH Madden unpublished PhD dissertation “Miocene Toxodontidae (Notoungulate, Mammalia) from Colombia, Ecuador and Chile” (Duke University). See Tables 2 and 3 for references. (TIF) [file pone.0156558.s001.tif]
