# Supplementary material for: Cranial Morphology of the Late Oligocene Patagonian Notohippid Rhynchippus equinus Ameghino, 1897 (Mammalia, Notoungulata) with Emphases in Basicranial and Auditory Region
Source: PLoS One. 2016 May 27;11(5):e0156558. doi: 10.1371/journal.pone.0156558 (PMC4883762; doi:10.1371/journal.pone.0156558)
Supplement: S1 Table — Afrotherian cranial synapomorphies listed by O’Leary et al. [3] and condition observed in R. equinus based exclusively on specimen MPEF PV 695. Numbers in parenthesis indicate character number in the original analysis. (PDF) [file pone.0156558.s002.pdf]

S1 Table: Afrotherian cranial synapomorphies and condition observed in MPEF PV 695

Cranial synapomorphies of Afrotheria according to O´Leary [3]	Condition observed in MPEF PV 695
(19) Vertical external nasal aperture in lateral view	Slanted nasal aperture in lateral view
(37) Nasal length equal or more than 40% of skull length	Nasals slightly shorter than 40% (38%) of skull length
(564) Absence of piriform fenestra	Piriform fenestra tentatively present
(671) Tensor tympani muscle fossa on tegmen tympani	? (Not enough ct resolution)
(818) Dorsal margin of MAE lower than highest point of ventral margin of zygomatic process	It matches MPEF PV 695 morphology
(911) Hypoglossal (condylar) foramen equidistant between jugular foramen and occipital condyle	Hypoglossal (condylar) foramen closer to occipital condyle than to jugular foramen
(972) Absence of sagittal crest	Sagittal crest present
(1012) Supraoccipital contributes to mastoid foramen	It matches MPEF PV 695 morphology
(1054) Presence of external occipital crest	Absent
(1125) Anteromedial orientation of IAM	Medial orientation of IAM
(1397) Rounded upper incisor root	It matches MPEF PV 695 morphology
(1765) Absence of metastyle in deciduous P4 (P3 in this paper)	? (No deciduous P3 in MPEF PV 695)
(1789) P4 (P3 in this paper) subequal to P5 (P4 in this paper)	Mesiodistal diameter of P4 (P3 in this paper) smaller than P5 (P4 in this paper)
(1790) P4 (P3 in this paper) area subequal to P5 (P4 in this paper)	P3 area smaller than P4 area
(1796) Presence stylar shelf in P4 (P3 in this paper)	Absent
(1819) Presence of hypocone in P4 (P3 in this paper)	Tentatively absent (difficult to appreciate considering lophodont condition and wear stage)
(1826) Presence of postcingulum in P4 (P3 in this paper)	Absent
(2319) Presence of preparacingulum on M3	Absent
(2341) M1 parastylar lobe (parastyle in this paper) strongly buccally oriented and hooklike (buccally concave)	Parastyle moderately developed and not hooklike
(2365) Subtriangular paracone and metacone shape with buccal face flat	? (not possible to appreciate considering lophodont condition and wear stage)
(2565) M2 talon well developed but not approaching size of the trigon	Notably smaller than trigon
(2603) Presence of hipocone in M3	Absent
