# Supplementary material for: Cranial Morphology of the Late Oligocene Patagonian Notohippid Rhynchippus equinus Ameghino, 1897 (Mammalia, Notoungulata) with Emphases in Basicranial and Auditory Region
Source: PLoS One. 2016 May 27;11(5):e0156558. doi: 10.1371/journal.pone.0156558 (PMC4883762; doi:10.1371/journal.pone.0156558)
Supplement: S2 Table — Laurasiatherian cranial synapomorphies listed by O’Leary et al. [3] and condition observed in R. equinus based exclusively on specimen MPEF PV 695. Numbers in parenthesis indicate character number in the original analysis. (PDF) [file pone.0156558.s003.pdf]

S2 Table: Laurasiatherian cranial synapomorphies and condition observed in MPEF PV 695

Cranial synapomorphies of Laurasiatheria according to O´Leary [3]	Condition observed in MPEF PV 695
(15) Alveolar line in a plane that intersects the glenoid fossa	It matches MPEF PV 695 morphology
(62) Dorsoventral thickness of slightly deeper than the length of the incisor roots	It matches MPEF PV 695 morphology
(190) Short facial process of the lacrimal	There is no facial process
(581) Epitympanic wing anterior to promontorium width equal or more than two thirds the width of the promontorium	Intermediate, less than two thirds the width of promontory
(746) Large stapedial fossa circumference, more than twice the circumference of fenestra vestibuli	? (Not enough ct resolution)
(771) Posterodorsal position of incisura tympanica	? (Ectotympanic contacts to surrounding elements are not distinguishable)
(852) Presence of muscular process of malleus	? (Not preserved)
(1044) Nuchal crest anterior to posterior surface of occiput	In line with posterior surface of occiput and slightly projected posteriorly
(1339) I1 spatulate (not conical)	It matches MPEF PV 695 morphology
(1429) C1 height similar to adjacent teeth	It matches MPEF PV 695 morphology
(1805) Absence of metacone on P4 (P3 in this paper)	Present? (inferred besed on the presence of metaloph)
(1814) Absence of protocone on P4 (P3 in this paper)	Present? (inferred based on the presence of protoloph)
(1930) Talon of deciduous P5 (P4 in this paper) notably smaller than the trigon	? (No deciduous P4 in MPEF PV 695)
(1945) area of P5 (P4 in this paper) subequal to M1	Area of P5 (P4 in this paper) is smaller than M1
(1979) Paracone of P5 (P4 in this paper) significantly higher than paracone of M1	not applicable considering lophodont condition and wear stage
(2378) Centrocrista of M1 extends barely above the level of the stylar shelf	not applicable considering lophodont condition and wear stage
